# Volume Changes of Alkali-Activated Slag-Based Mortars and Concretes in Sealed and Free Conditions

**DOI:** 10.3390/ma18194577

**Published:** 2025-10-02

**Authors:** Maïté Lacante, Brice Delsaute, Stéphanie Staquet

**Affiliations:** BATir Department (LGC), Université Libre de Bruxelles (ULB), 1050 Brussels, Belgium; maite.lacante@ulb.be (M.L.); brice.delsaute@ulb.be (B.D.)

**Keywords:** mortar, concrete, coefficient of thermal expansion, autogenous strain, heat flow, alkali-activated materials, blast-furnace slag, sodium hydroxide

## Abstract

The goal of this paper is to assess the evolution of the autogenous strains as well as the thermal strains (thanks to the assessment of the coefficient of thermal expansion) of alkali-activated slag-based materials at early age. The effect of the sand and the coarse aggregates on the paste and mortar scale to upscale to mortar and concrete, respectively, has been investigated as a function of the age of the material. The restraint imposed by the sand on the paste seemed more significant than that of the coarse aggregate on the mortar. In addition, the long-term autogenous strains have been monitored on the mortar scale. These results revealed a separation into groups based on the solution concentration. Different testing methods were also compared. Thermal and autogenous strains were monitored with a customized testing device where the thermal variations are controlled. These devices were the horizontal corrugated tubes method (for tests on paste and mortar scales) and the vertical corrugated tubes method (for tests on mortar and concrete scales). Depending on the compositions (lower concentration), good correlations can be obtained between the two testing methods. Moreover, the autogenous strain of two different specimen sizes was also assessed manually (initially for the long-term), but early-age comparison showed good correlation for lower solution-to-binder ratios. On the concrete scale, a correlation based on the modified equations from the standards was established between the compressive strength and the tensile strength, obtained from the splitting tensile test.

## 1. Introduction

As it is widely known, the production of Portland cement accounts for approximately 5 to 8% of the worldwide CO_2_ emissions by humans, depending on the sources. Therefore, it is very important to promote development and research into sustainable alternatives [1,2,3,4]. One of the strategies to reduce this environmental impact is the use of different materials. Therefore, interest is currently being directed to the development and the adoption of alternative materials having a lower associated footprint and produced from industrial by-products, such as alkali-activated materials [5,6,7,8,9]. Alkali-activated materials are typically obtained by mixing a precursor (blast-furnace slag, fly ash, metakaolin, …) to an activator (sodium hydroxide, sodium silicate, sodium sulfate, …) [8,10,11,12,13,14]. The properties and performances of alkali-activated materials strongly depend on the composition and its internal parameters [15,16,17,18,19].

The autogenous strain in AAM typically consists of two stages (when chemical shrinkage is not considered) [20], similar to those observed in PC-based compositions [21]. First, a swelling can be observed, although this is not always the case. Swelling-responsible reaction products are formed during the reaction. These are hydrotalcite group minerals [22] and act similarly to the formed ettringite in PC-based materials [23]. In addition, the swelling can be caused by the increase of internal relative humidity during the reaction [24]: this rise in internal relative humidity leads to a pressure reduction because of the increase of the pores’ Kelvin radii, and thereby swelling occurs. Another contribution to the swelling, particularly observed in alkali-activated or cementitious materials with higher solution-to-binder ratio (S/B) or water-to-cement ratio (W/C), is the reabsorption of the solution following the chemical shrinkage, which causes an increase of the swelling [23]. The second stage is shrinkage (commonly called self-desiccation shrinkage in PC-based materials). The evolution of the pore structure leads to an increase of the surface tension. In addition, this effect is typically more pronounced in AAM than in PC-based systems [22]. The reaction produces C-A-S-H gels, which are highly viscous and increase the deformability of AAM compared to PC [22,25]. Moreover, during the reaction and consequently the solid network formation, polycondensation takes place between the different gel units. This leads to a decrease of the interparticle distance, thereby resulting in shrinkage [26]. A last contribution to the shrinkage component of the autogenous strain is linked to the imbalance of the forces. During the progression of the reaction, the concentration of ions in the pore solution decreases over time and over the degree of reaction [20,27]. Consequently, the steric hydration forces are reduced. These are repulsive. On the other hand, between the same gel particles, the opposite (attractive) forces are not impacted and remain unchanged [28]. As a result, shrinkage is obtained since the constant (non-changing) attractive forces are countered by diminishing repulsive forces.

The autogenous strain is closely linked to the internal parameters of the paste. An increase of the solution-to-binder ratio typically results in lower shrinkage (because of its mitigating effect on self-desiccation) and in an increase of the swelling (because of the presence of more liquid in the system). On the other hand, increasing the concentration of the solution results in higher shrinkage (increased self-desiccation process) and reduced apparent swelling (because of the overlap of swelling and shrinkage) [29].

The inclusion of sand and aggregates in the matrix reduces the overall strain [21]. On one hand, it might be related to the replacement of a part of the paste (which shrinks) by sand or aggregates (which do not shrink). Therefore, the overall shrinkage might be reduced. On the other hand and considered as most important effect, is the restraining action of the sand or aggregates on the strain of the paste [22,30,31].

Previous research has shown that alkali-activated pastes made from blast furnace activated by sodium hydroxide solution can exhibit higher autogenous strain than PC-based materials [20]. The results strongly depend on the amount of slag, the water content and the alkali content in the paste. This is also the case for the thermal strains, characterized by the coefficient of thermal expansion.

The swelling seems to be more present in the case of mortar than in paste [32,33]. The autogenous shrinkage of paste (metakaolin-fly ash activated by sodium hydroxide and sodium silicate) can be reduced from 123–480 µm/m to 87–294 µm/m by adding sand and thus upscaling to mortar, as described by Yang et al. [32] where the sand-to-binder ratio was 0.5 and 1.0. On the other side, higher strains were also reported: approximately between 1427 and 2610 µm/m around the same age for mortars made from blast-furnace slag activated with sodium silicate and a binder-to-sand ratio of 0.5 [33], or approximately between 2400 and 4500 µm/m for blast furnace slag activated by 8 M sodium hydroxide and sodium silicate with a solution-to-binder ratio of 0.4 and a sand-to-binder ratio of 1.25 [34] compared to an autogenous shrinkage of about 500 µm/m for a PC mortar with similar composition characteristics. This indicates quite different strain ranges for AAM, strongly depending on the composition characteristics. The addition of sand is responsible for the reduction of the autogenous shrinkage [22,32], similar to PC-based materials [21]. This effect is due to the restraining role of the sand on the paste, and not because of the dilution of the binder when sand is added into the mix [22].

The multiscale behavior (mortar and concrete) of AAM is studied on this paper. The paste scale has already been investigated in Lacante et al. [20]. Additionally, different testing methods are evaluated at the mortar scale to determine their consistency and whether they provide comparable results. Then, the long-term autogenous strain of the compositions is assessed. Finally, the behavior at the concrete scale is investigated. Unlike the mortar and paste scale, the concrete scale remains a relatively poorly investigated topic in the field of alkali-activated materials behavior.

This research paper aims to investigate the early-age autogenous and thermal strains of the paste-mortar-concrete upscaling because of the presence of limited investigations into these concrete-scale investigations as well as the lack of transitions between the different scales from a point of view of volume changes in free and sealed conditions. This paper offers a multi-technique investigation into the three different scales using the same materials and internal parameters when changing scales. For this, different testing techniques have been used for the study of the autogenous strains, the thermal strains and the reaction kinetics. Finally, the autogenous strains were investigated at early-age and on the long term in order to assess how the autogenous strain might contribute to the overall behavior of the material. This work offers a multi-technique investigation into the multi-scale volume changes of alkali-activated slag.

## 2. Materials

### 2.1. Raw Materials

#### 2.1.1. Blast-Furnace Slag

The precursor used in this paper is blast-furnace slag. The slag has a density of 2.87 g/cm^3^ and a Blaine fineness of 469 m^2^/kg. The chemical composition of the slag can be found in Table 1. These characteristics have been determined in accordance with the European standard EN 196-6 [35] by CRIC-OCCN [36], the Belgian collective research center for the cement sector and the sector of ready mix concrete.

#### 2.1.2. Sodium Hydroxide

The activator used in the paper is sodium hydroxide (NaOH), from VWR (Leuven, Belgium). NaOH has a molar mass of 40 g/mol. The solution is obtained by dissolving NaOH pellets in deionized water using a magnetic stirrer, from VWR (Leuven, Belgium). The purity of the NaOH pellets is 97%. Two different concentrations have been investigated: 2.0 mol/L and 8.0 mol/L [20]. In addition, two different solution-to-binder ratio are investigated: 0.5 and 0.8 [20]. Once prepared, the solutions are maintained in sealed containers at (20 ± 2) °C in the lab for at least 24 h before mixing.

#### 2.1.3. Sand Aggregates

Two types of sand have been used throughout this research in the mortar compositions. Both sands have been briefly compared in Section 4. Both sands are 0/4 river sands. Sand 1 was provided by Lafarge Granulats France (Sandrancourt, France) (absorption coefficient of 1.36%) while Sand 2 was provided by CRIC-OCCN (Brussels, Belgium) (absorption coefficient of 0.1%).

The sand is oven dried at 105 °C during 24 h. Afterwards, it is stored in a sealed container such that it can cool down in a closed environment.

#### 2.1.4. Coarse Aggregates

For the concrete compositions, three different types of coarse aggregates (nature: Calcaire Tournaisis) have been used in addition to the sand. These coarse aggregates were provided by CRIC-OCCN (Brussels, Belgium). The smallest aggregates were 6.3/10, the medium aggregates were 10/14 and the biggest aggregates were 14/20. They all have an apparent density of 2.65 kg/m^3^ and an absorption coefficient is 0.6%.

Similarly to the sand, the coarse aggregates were oven dried at 105 °C during 24 h. Afterwards, they were stored in separate sealed containers such that they could cool down in a closed environment without being mixed together.

### 2.2. Compositions

This paper investigates the volume changes of the alkali-activated slag at the mortar scale and concrete scale. The mortar compositions are presented in Table 2, where S/B is the mass solution-to-binder ratio and W/B is the mass water-to-binder ratio. The binder refers to the amount of slag present in the paste. The mortar specimens were prepared using a mass paste-to-sand ratio of 1. Using a higher paste-to-sand ratio, as seen for PC mortars, would have been challenging because of the loss of workability for the 0.5 S/B compositions, increasing the casting difficulty. For comparison with paste-scale behavior, the results presented in Lacante et al. [20] will be referenced. Concerning the choice of the solution-to-binder ratio, lower values would have resulted in poor workability, which could impact the casting time as well as the quality of the casting of the samples. Higher S/B increase the bleeding risk. Similarly, for the concentration of the alkaline activating solution, two values were chosen. These are interesting and realistic concentrations in this field. Lower values result in lower reaction as well as increased bleeding risk, while higher values become more difficult to prepare and result in high and fast loss of workability over time.

The effective solution-to-binder and effective water-to-binder ratios resulting from the absorption of liquid by the sand have been evaluated. This yielded respectively 0.48 and 0.44 for M-S05M2, 0.48 and 0.35 for M-S05M8, 0.78 and 0.72 for M-S08M2 and 0.78 and 0.58 for M-S08M8, for Sand 1. These parameters for Sand 2 remained equal to the S/B and W/B in Table 2 (the highest difference was 0.41%). This yields an average difference of (3.82 ± 0.83)% between the two sands and a difference of (4.12 ± 0.90)% between the non-effective ratio and the effective ratios resulting from Sand 1.

Compositions M-S05M2, M-S08M2, and M-S08M8 were selected for upscaling from mortar to concrete. Composition M-S05M8 was not upscaled to concrete due to its poor workability observed at the mortar scale, see Section 4. The concrete mix design was based on an ordinary Portland cement-based concrete. Even though an initial literature review was conducted for the design of alkali-activated concrete, limited detailed information was available. In most cases, only general mix proportions were reported. Occasionally, a distinction was made between sand and coarse aggregates [37,38,39,40], but specific proportions per aggregate size were rarely shared [41].

The consistency as well as vibration capacity were assessed visually. Based on these evaluations, the selected compositions were then tested for compressive strength. The details of the mix designs for the concrete compositions, as well as the granular skeleton and the water content of the solution, are presented in Table 3. These quantities were selected to produce approximately 3.5 L of concrete. The mix design maintained the same key characteristics as at the mortar scale: a solution-to-binder (S/B) ratio of 0.5 or 0.8, a molar concentration of 2 M or 8 M, and a paste-to-sand ratio of 1. The volumetric ratio of the volume of coarse aggregates (6/10, 10/14 and 14/20) over the volume of mortar is 0.557 for each composition.

The effective solution-to-binder and effective water-to-binder ratios resulting from the absorption of liquid by the sand and the aggregates have been evaluated. This yielded respectively 0.49 and 0.45 for M-S05M2, 0.78 and 0.73 for M-S08M2 and 0.78 and 0.59 for M-S08M8. This yields an average difference of (2.14 ± 0.35)% between the non-effective ratio and the effective ratios.

## 3. Testing Devices and Procedures

### 3.1. Preparation and Mixing

Before the mixing, the materials were cured during at least 24 h at the intended curing temperature for the test that was planned. Therefore, the materials were cured in the lab at (20 ± 2 °C). Right after the mixing is done, the temperature of the material is measured to ensure proper temperature control and to accurately estimate the maturation of the material.

#### 3.1.1. Mortar

The mixing of mortar has been performed in two different types of mixers. Depending on the mixing device, the mixing procedure changed. The mixing procedure was based on the mortar mixing procedure detailed in European standard EN 196-1:2016 [42] and was performed using a 1 L Hobart mixer, from 3R Labo (Saint-Rémy, Belgium). The activating solution was poured into the pre-humidified bowl. The precursor and the sand are added. The mixer is started at low speed (140 ± 5 rpm) for 60 s, followed by 30 s of high-speed mixing (185 ± 10 rpm). Next, the mixer was stopped, and the paste was allowed to rest for 90 s, during which the bowl was scraped with a rubber scraper to remove any material adhering to the sides or the bottom and added in the middle of the mixture. Subsequently, the materials were mixed for an additional 60 s at high speed. Finally, the mix was poured into molds, which were then sealed and placed in a climatic chamber for curing or into the appropriate device for testing.

When the mixing was done in the 12 L mixer, the beginning of the procedure changed. First, the mixer was slightly humidified and the sand and the precursor were added together, as prescribed by the European standard EN 480-1:2023 [43]. The mixer was closed and was launched during 30 s at low speed. The door was then opened to add the alkaline solution. Next, it was closed again. Then, it followed the 1 L mixing protocol.

#### 3.1.2. Concrete

The mixing procedure for the concrete specimens is based on the European standard EN 480-1:2023 [43]. Two sets of testing campaigns were also conducted for the concrete scale. The compositions for the preliminary testing campaign were prepared in a smaller 5 L Hobart mixer. The compositions for the in-depth testing campaign were prepared in the 12 L mixer. All the coarse aggregates and the sand as well as half of the activating solution were added in the pre-humidified mixer. The mixer was started at low speed during 120 s. The mixing was paused during 120 s. The precursor was added in 30 s. After which, the other half of the solution was added to the mix in another 30 s. The mixer was started again and mixed the materials during 120 s.

The Hobart mixer was covered with a cloth during the mixing and the pausing to minimize the evaporation effects.

### 3.2. Compressive Strength

The compressive strength of the mortar specimen was evaluated by testing cubes (50 mm side).

As per previous works [21,44], the average of two cubes is presented for each composition at each age (i.e., 1, 3, and 7, as well as 28 days). As required by the ASTM C109 standard [45], the range between specimens was less than 7.6%.

The tests were conducted on a hydraulic Galdabini press of 600 kN with a sensitivity of 1 kN following the ASTM C109 standard [45], which stipulates that the loading rate should range between 900 and 1800 N/s. Moreover, this rate must be obtained within the first half of the expected maximum load and no adjustments are allowed later.

The concrete samples were tested with cubes of 10 cm a side. Similarly to the 5 cm-side cube test, two specimens were tested per age following the same repeatability criterion. A conversion factor between two sizes of samples presented in this section exists for PC-based concretes. This factor is 0.935 [21].

### 3.3. Splitting Tensile Test

The splitting tensile test was conducted on the concrete compositions according to the ASTM C496 standard [46]. For this purpose, 16 cm by 32 cm cylindrical specimens were cast. Initially, two samples per testing age (i.e., 1 day, 3 days and 7 days) were cast as well as an extra sample in case the repeatability was not good. However, the repeatability was satisfactory for each test in compliance with the standard. Therefore, the extra sample was tested at 28 days. A constant loading rate is applied until failure.

At the end of the test, the maximum load *F* [N] is obtained. The tensile strength $f_{t}$ [Pa] can be deduced and is considered about 10% lower than the obtained splitting tensile strength $f_{ts}$ [Pa], see Equation (1) where *D* is the diameter of the specimen [m] and *L* is the length of the specimen [m] [46].

(1) $$f_{t}=0.9\;\cdot \;f_{ts}=0.9\;\cdot \;\frac{2\;\cdot \;F}{\pi \;\cdot \;D\;\cdot \;L}$$

### 3.4. Apparent Density

The apparent density was computed on the basis of the weight and the dimensions of the cubes intended for the compressive strength tests, at the same ages. The densities reported in Section 4 and Section 5 correspond to the average of all measurements for one composition because no consistent trend was identified over time.

### 3.5. Isothermal Calorimetry

The (exothermic) reaction process of the materials was studied using the TAM Air isothermal calorimeter (from TA Instruments, New Castle, DE, USA) [47,48], which satisfies the European norm EN 196-11:2018 [49].

This calorimeter comprises eight channels which can be used simultaneously and separately, although at the same temperature. Each of these channels has two spots. The first one serves to monitor the ampoule filled with about 7.5 g of the material that must be tested. The second spot contains an inert reference ampoule filled with sand in this case, to which the tested ampoule’s heat flow is compared. Each spot is equipped with its own heat flow sensor. The heat flow from the tested sample is directly compared with that of the inert reference thanks to this twin configuration, thus reading the noise and increasing the measuring stability. This allows for erasing noise, which also increases the stability of the measurement [48]. The device is calibrated after each temperature change and at the start of each testing campaign. The ex-situ mixing was used. The investigated mixture is taken from a mixing prepared for the other tests in this study, allowing the tests to be conducted in parallel. An ampoule is filled with paste, sealed and placed into the calorimeter within the first ten minutes after the mixing has started. Two samples of each composition are monitored for 14 days. In addition, the repeatability criterion suggested by the corresponding standard was respected.

The measured heat flow exhibits two peaks. The first peak characterizes the early dissolution of the slag and is often missed because of its very early occurrence and the ex-situ mixing. This is linked to the reactants dissolution (breakdown of bonds in the slag), the particles wetting and, finally, the formation and interaction of units of silicate with Ca^2+^ and Na^+^ ions [50,51,52]. Then, the second peak is monitored. This is related to the formation of the reaction products (i.e., calcium aluminosilicate hydrates) [50].

The TAM Air isothermal calorimeter serves to follow the heat release of pastes and mortars. However, it can not be used for the concrete scale as the ampoules are too small compared to the aggregates sizes. Therefore, interest has been given to the Quasi-adiabatic calorimeter for the concrete compositions and as a consequence the mortar compositions to perform an optimal analysis, see Appendix A.

### 3.6. Early Age Autogenous Strain and Coefficient of Thermal Expansion with Corrugated Tubes Method

The corrugated tubes methodology is based on the AutoShrink [53] method outlined in ASTM standard C1698–09 [54], which has been adapted to study thermal strains in parallel, and thus the coefficient of thermal expansion [44,55].

Its primary purpose is to monitor the (linear and unrestrained) autogenous strain of cementitious materials cured under sealed conditions during the hardening process. Special corrugated plastic molds (40 cm in length and 30 mm in diameter) prevent moisture loss while allowing the specimen to shrink without being restrained longitudinally due to the lowered longitudinal stiffness of the tubes, thereby meeting the sealed conditions. Following the testing protocol and casting time, the deformation of two samples per composition is monitored, in addition to the internal temperature of a third sample. Distinguishing the thermal strain from the autogenous strain can be difficult because both parameters evolve at the same time. Therefore, the customized AutoShrink at ULB [55] is equipped with a thermal regulation around the rigid frame to apply repeated temperature variations (variations of ±3 °C around the curing temperature (20 °C). The thermal variations are as small as possible and are applied as fast and short as possible in order to avoid any (irreversible) creep-like contributions that arise from delayed thermal strains [56]. In the present case, the hypothesis that the thermal strains evolve linearly with the temperature is valid.

Since the repeated temperature variations are applied to the entire set-up, the rigid frame undergoes thermal deformation as well. Consequently, the temperature inside the AutoShrink is recorded in addition to the internal temperature of the third sample.

At a very early age, it is necessary to decouple the strains, whereas after a certain period of time, the autogenous strain can be considered constant over a small time range, which corresponds to the interval in which the CTE at that time is computed. Therefore, CTE and the autogenous strains are calculated approximately every 2 h. The decoupling procedure has been established by Delsaute and Staquet [55,57,58]. In summary, this procedure implies considering a sample cured at 20 °C (without any thermal variations). This is obtained by applying a cubic interpolation on the measured strain at the ages where the temperature is 20 °C and when no thermal gradients remain in the tested specimen. The experimental thermal strains are obtained by subtracting the previously computed strain from the total experimentally measured strain. The coefficient of thermal expansion can be computed by considering that the thermal strain is the product of multiplying the coefficient of thermal expansion by the temporal temperature variation applied to the material. Consequently, the experimental autogenous strains could be calculated. Therefore, CTE and the autogenous strains are computed about every two hours.

The autogenous strain results were initialized with the knee-point method [29,59]. The coefficient of thermal expansion results are presented from the first physically realistic data point (0 µm/m/°C < CTE < 100 µm/m/°C) on. Higher early CTE values might not only represent free strain as material sedimentation and settling occur before setting [57]. The average autogenous strain and average coefficient of thermal expansion of two samples and the minimum and maximum values are reported in Section 4. This method was used to investigate the autogenous and thermal strain of the mortar compositions to compare to those of the corresponding paste compositions obtained with the same method.

### 3.7. Early-Age Autogenous Strain and Coefficient of Thermal Expansion with BTJade Method

The evolution of the autogenous strain and coefficient of thermal expansion of concrete can not be monitored with the corrugated tubes method. Subsequently, interest was also given to a second automatically continuous method: the BTJade (“BéTon au Jeune Age, déformations endogènes” which means “Concrete at early-age, autogenous strains”) test rig established by Claude Boulay [60]. The test rig consists of a (vertical) corrugated mold and fixed metal parts. The whole set-up is placed in a water tank. The temperature inside the water tank is controlled by a thermostatic bath.

In contrast to the corrugated tubes method, a thermocouple is present in each sample [60]. Therefore, during the test, the vertical displacement of the specimen, the specimen’s temperature, the temperature inside the water tank as well as the temperature above the tank cover are measured. Following the testing protocol and casting time, the deformation of two samples per composition is monitored.

In this case, the PVC corrugated tube has a diameter of 125 mm and the length of the sample is about 225 mm.

After the test, the influence of the temperature on the testing rig inside the water tank as well as the room temperature on the displacement sensor which is not in contact with the water is taken into account in Equation (2) [60]. That is why the temperature above the cover was also monitored. Finally, the total strain $\epsilon_{tot}$ corresponding to the sum of the autogenous strain $\epsilon_{auto}$ and the thermal strain $\epsilon_{thermal}$ is obtained.

(2) $$\epsilon_{tot}=\epsilon_{auto}+\epsilon_{thermal}=\;\epsilon_{auto}+\alpha_{CTE}\;\cdot \;\Delta T=\frac{\left(C_{Tec}\;\cdot \;\Delta T_{ec}+C_{Tsc}\;\cdot \;\Delta T_{sc}-\Delta l_{m}\right)}{L_{0}}$$

where

- $T_{ec}$ is the temperature of the tank water [°C].
- $T_{sc}$ is the temperature of air above tank cover [°C].
- $C_{Tec}$ is the thermal coefficient relating the displacement sensitivity to the changes in temperature present in the tank water [m/°C].
- $C_{Tsc}$ is the thermal coefficient relating the displacement sensitivity to the changes in temperature present above the tank cover (near the displacement sensor) [m/°C].
- $L_{0}$ is the base length at the end of the test.
- $\Delta l_{m}$ is the displacement between age *t* en age $t_{0}$ measured by the displacement sensor, where $t_{0}$ is the age at which the first data point of the test was recorded.
- ΔT is the (temporal) temperature variation in the material.
- $\alpha_{CTE}$ is the coefficient of thermal expansion (CTE) of the material.

Decoupling between the autogenous strains and the thermal strains is also necessary in this test. This is done in the same way as explained for the corrugated tubes method [55,57]. The results of the autogenous strain were initialized with the knee-point method [29,59]. The results of the coefficient of thermal expansion are presented starting from the first physically plausible data point (0 µm/m/°C < $\alpha_{CTE}$ < 100 µm/m/°C). The average strain and coefficient of thermal expansion of two samples and the minimum and maximum values are reported in Section 4 and Section 5.

This device has been used to monitor the autogenous strains and the coefficient of thermal expansion of mortar and concrete samples. A part of the results will be used to assess the repeatability of the results between the corrugated tubes method and the BTJade method on the mortar scale.

### 3.8. Equivalent Age and Maturity Concept

The results at early-age for the autogenous strain and coefficient of thermal expansion are presented as function of the equivalent age in order to take into account the maturity changes that could occur due to internal (reaction process) and external (imposed temperature cycles) thermal changes. Therefore, the equivalent age is computed with the apparent activation energy determined for each composition on the paste scale with isothermal calorimetry, see Lacante et al. [20].

### 3.9. Early-Age and Later Age Manual Monitoring of the Autogenous Strain with the DEMEC

#### 3.9.1. Monitoring on Small Samples

The autogenous strain has been monitored manually with the DEMEC mechanical strain gauge. A 20 cm DEMEC device was used to monitor the autogenous strain. This manual measurement method allows for a long-term monitoring of samples compared to automatic methods such as the corrugated tube method and is based on the ASTM standard C 490/C 490M-08 [61]. After mixing, the material was poured into the molds measuring 28.5 cm × 2.5 cm × 2.5 cm (internal measurements). For this test, three samples were cast. Once the samples were demolded, they were sealed with two layers of self-adhesive aluminum foil. Two thin metal measuring discs with a blind hole were glued on each of the four sides of the samples, with a 20 cm distance between them. The blind holes allow the positioning of the measuring part of the device.

The DEMEC method was used to monitor the autogenous strain at early age and on the long term. The early-age monitoring allowed to compare the results with the corrugated tubes method. This method was used for the mortar samples. These results are compared to those on the paste scale, obtained with the same method [20], as well as with the early-age strain obtained with the corrugated tubes method. This allows to compare several testing techniques.

#### 3.9.2. Monitoring on Big Samples

Similarly to the two sizes of specimens tested for the automatic and continuous monitoring of the autogenous strains, two samples sizes have been tested for the mortar compositions. For this second method, three 10 cm × 10 cm × 40 cm specimens were cast per composition in order to follow the European standard EN 12390-16:2019 [62]. This time, it was chosen to work with a 15 cm DEMEC device to monitor the specimens as it provided a higher accuracy. Therefore, the thin metal measuring discs were glued at a distance of 15 cm from each other.

The results obtained with this method on the mortar scale are compared to the results obtained with the small DEMEC method as well as with the BTJade method (at early-age) for the same scale. This allows to assess the repeatability between different testing methods.

## 4. Results and Discussion on the Investigation of the Upscaling from Paste to Mortar

### 4.1. Apparent Density

**Figure 1:**
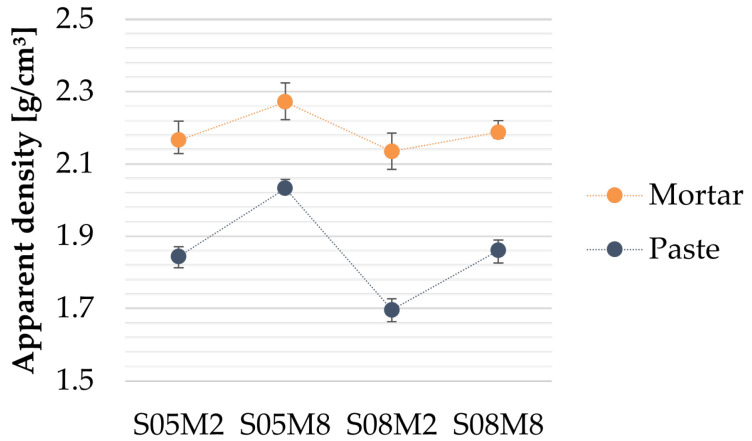
Apparent density of AAS mortars.

The apparent densities of the paste and mortar compositions are compared in Figure 1. As expected, the density increases for each composition with the addition of sand [63], due to the higher density of sand (2.52 g/cm^3^) relative to the paste. A similar trend is observed across all compositions when compared at the paste scale. However, the overall standard deviation between compositions is reduced at the mortar scale.

### 4.2. Compressive Strength

**Figure 2:**
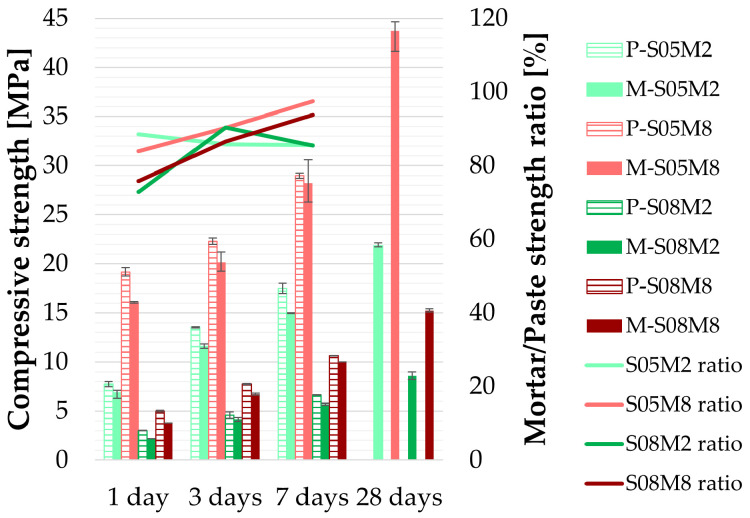
Compressive strength of AAS mortars and ratio between compressive strength of mortar and paste, as a function of the age.

The compressive strength of AAS mortars is compared to that of the corresponding pastes in Figure 2. In all cases, the mortar exhibits lower compressive strength than the paste [64]. Conversely, Klun et al. [63] and Wei et al. [17] obtained higher compressive strength for mortar than for paste. This is closely related to the bonding effect between the aggregate and the cementitious material.

On average, the compressive strength of mortar is at least 72% of that of the corresponding paste (M-S08M2). With increasing age, the strength ratio between mortar and paste increases for the 8M compositions, reaching up to 97% for M-S05M8. In contrast, for M-S08M2, the ratio fluctuates with time, while for M-S05M2, a slowly decreasing trend is observed.

Despite the lower initial strength of mortars, a substantial increase of 51.0 (±3.2)% in compressive strength is observed between 7 and 28 days, related to the ongoing reaction and strength development.

### 4.3. Isothermal Calorimetry

#### 4.3.1. Heat Flow and Cumulative Heat

The reaction kinetics were first monitored using isothermal calorimetry.

**Figure 3:**
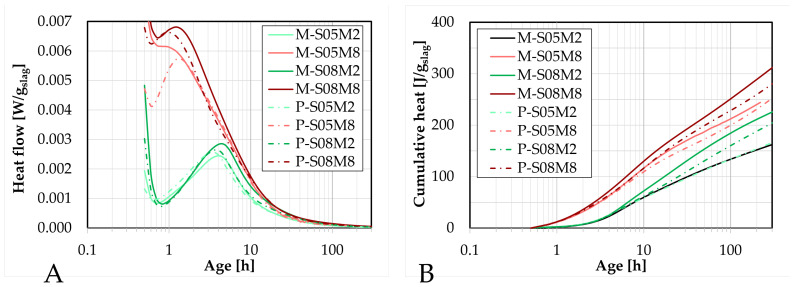
Results of isothermal calorimetry on mortar scale compared to the paste scale: (**A**). heat flow, (**B**). cumulative heat.

The results of the isothermal calorimetry tests are presented on Figure 3, and are normalized per gram of slag. A small but distinguishable difference can be observed between the results on mortar and on paste in terms of both heat flow and cumulative heat. Specifically, mortars exhibit a slightly higher heat flow, resulting in a higher cumulative heat release compared to pastes.

At early age, the cumulative heat curves for specimens activated with the same molar concentration are initially superimposed, consistent with previous observations that the S/B ratio does not significantly influence the very early-age cumulative heat release [20]. However, as the reaction progresses, these curves begin to separate. This divergence occurs earlier in mortar specimens, while the paste curves remain superimposed for a longer duration. This indicates that the presence of sand might accelerate the influence of the S/B ratio on cumulative heat release, and by extension, on the heat flow.

For the S05M2 composition, the cumulative heat curves for paste and mortar are nearly identical, indicating that the sand is inert, as expected. In contrast, for other compositions, small deviations are observed, suggesting that sand may result in a minor influence on reaction kinetics. Nevertheless, the addition of sand does not significantly change the overall behavior of the heat flow curves or the observed effects of S/B ratio and molar concentration. Overall, sand has only a minimal impact on the reaction rate [23].

A lower S/B ratio appears to reduce the influence of sand on reaction kinetics. This may be attributed to the increased risk of segregation at higher S/B ratios.

Since sand has a certain absorption coefficient, it is possible that it absorbs a small part of the alkaline solution, thereby slightly decreasing the effective S/B ratio. However, this effect does not concur with observations from the study on the paste scale. It was shown that reducing the S/B ratio in paste leads to a slight decrease in both heat flow and cumulative heat [20]. This trend is not observed in the mortar specimens under the absorption hypothesis.

Another possible factor is the homogeneity of the mix. An ex-situ mixing approach was adopted to allow parallel testing, but this method might introduce small uncertainties in component quantities. Higher S/B ratios increase the likelihood of segregation, even immediately after mixing, in the mixing bowl. Despite efforts to obtain a representative sample poured in the ampoule to be tested in the calorimeter, it is possible that a higher amount of paste relative to the sand quantity is collected. As sand has a greater density, it tends to sink during segregation, potentially resulting in a locally increased paste-to-sand ratio. This would increase the slag content in the sample and could explain the slightly higher heat release observed in some mortar compositions. Moreover, this explanation aligns with the observation that the difference between the paste and the mortar samples becomes more pronounced at higher S/B ratios, which increases the segregation risk.

Nevertheless, this effect reflects a realistic scenario that can be encountered during the preparation and the handling of the material. Therefore, the results are presented as a valid representation. In practice, achieving perfectly homogeneous mixtures is unlikely, and small variations such as these may happen.

#### 4.3.2. Degree of Reaction and Ultimate Heat

Due to the observed differences in cumulative heat between paste and mortar scales, the ultimate heat release $Q_{\infty }$ was reassessed for the mortar compositions. The ultimate heat was estimated using both polynomial and exponential fitting methods, as described in [65].

**Figure 4:**
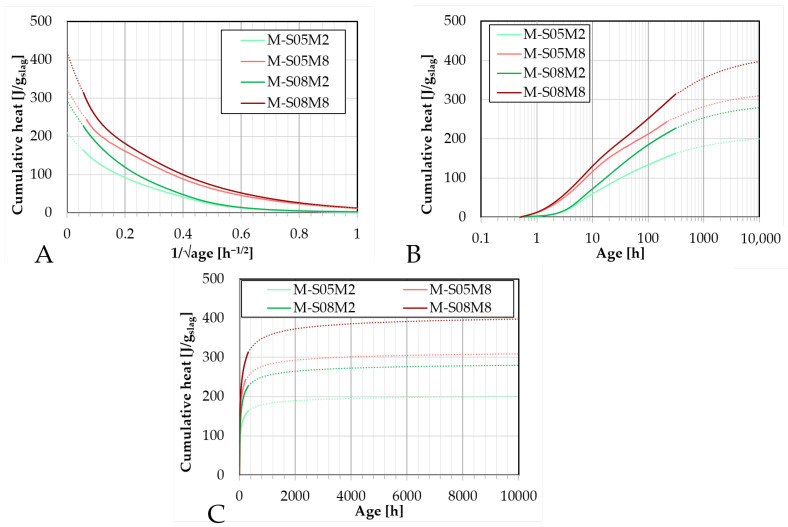
Fitting and extrapolation results (dotted lines) of AAS mortar to determine the ultimate heat based on the cumulative heat results (full lines): A. as function of the inverse of the square root of the age; B. as function of the age (logarithmic scale); C. as function of the age.

The results of the polynomial extrapolations are presented in Figure 4, while the corresponding fitting parameters are provided in Table 4.

A maximum difference of 13.6% (with an average 7.7 (±3.5)%) was observed between the ultimate heat values computed from the mortar and the paste scale results.

For the compositions with an S/B ratio of 0.5, the ultimate heat of the mortars is slightly lower than that of the corresponding pastes. Conversely, for compositions with an S/B ratio of 0.8, the ultimate heat is higher for mortars compared to pastes.

The ultimate heat values determined on paste using the exponential fitting method were 295.33, 417.61, 327.32, and 474.26 J/g for P-S05M2, P-S05M8, P-S08M2, and P-S08M8, respectively. In all cases, the ultimate heat values obtained for mortars were slightly lower than those for the corresponding pastes, see Table 5. The maximum difference observed between mortar and paste was 26.8% (with an average difference of 9.77 (±11.8)%), which is higher than the differences observed using the polynomial fitting method.

Overall, the exponential method yielded higher ultimate heat values than the polynomial approach. On average, an increase of 13.3 (±11.0)% was observed between the two methods at the mortar scale, although this increase is less pronounced than that reported at the paste scale (35.27 (±6.55)%).

The degree of reaction (DOR) can then be computed as the ratio between the cumulative heat at time *t* over the ultimate heat determined by one of the methods. The latter will be compared in the next sections.

#### 4.3.3. Apparent Activation Energy

In order to account for the effect of temperature on the maturity of the material, the activation energy used in this paper is the same as that determined for the paste scale, see Lacante et al. [20].

### 4.4. Early-Age Autogenous Strain

The autogenous strain was evaluated at both early and later ages. At early age, two different measurement techniques were investigated and compared: the corrugated tube method, representing a smaller-scale approach, and the BTJade method, which operates at a larger scale. The motivation for using and comparing two early-age testing methods was to assess the upscaling of autogenous strain behavior. The corrugated tube method is suitable for both paste and mortar scale investigations, but it cannot be applied to the concrete scale investigations. Therefore, a second technique was implemented to allow a reliable upscaling from mortar to the concrete scale. Additionally, the comparison allows for an assessment of whether these methods yield comparable results for alkali-activated materials, as it was the case for PC-based materials [21,55].

#### 4.4.1. Corrugated Tubes Method

**Figure 5:**
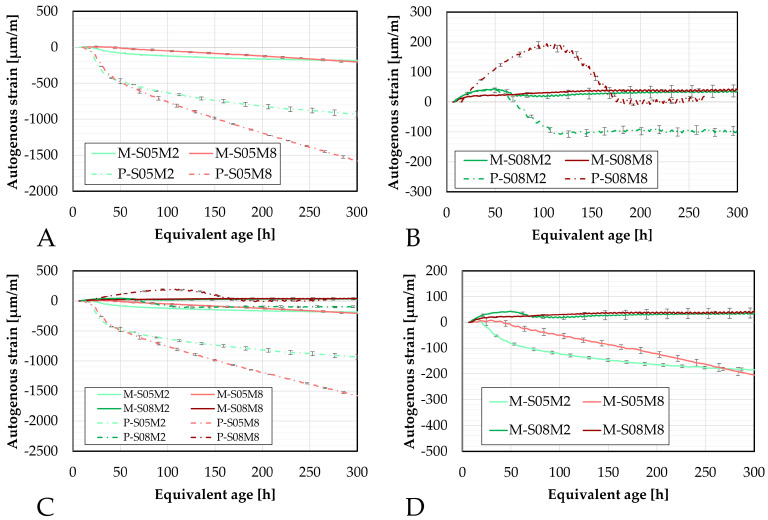
Autogenous strain of AAS mortars obtained with the corrugated tubes method, presented as a function of the equivalent age: (**A**). Compositions activated with S/B = 0.5 (compared to the equivalent paste compositions), (**B**). Compositions activated with S/B = 0.8, (**C**). Comparison of all mortar and paste compositions, (**D**). Comparison of the mortar compositions. M = mortar, P = paste.

The first method investigated is the corrugated tube method, which was also directly compared to paste scale results as a function of equivalent age, see Figure 5. As expected, the addition of sand significantly reduces the autogenous shrinkage. For the S/B = 0.5 compositions, the shrinkage component is significantly decreased, while the apparent swelling is increased. Since sand is chemically inert, it does not contribute chemically to the reaction. This reduction in shrinkage might allow the swelling effect to become more predominant in the total strain observed. Therefore, the increased apparent swelling possibly reflects a reduced overlap between the two competing phenomena, allowing swelling to become more visible as the shrinkage is reduced.

An exception to this trend is M-S08M8, which shows reduced swelling without the appearance of later-on shrinkage as it could be observed on the paste scale. Interestingly, the swelling of M-S08M2 follows closely that of its corresponding paste (P-S08M2), suggesting that in this case, the addition of sand has minimal impact on the swelling phase. Moreover, the apparent swelling is not increased, indicating that at higher S/B ratio, the swelling and shrinkage mechanisms are less likely to overlap, or at least not as much, compared to lower S/B compositions.

**Figure 6:**
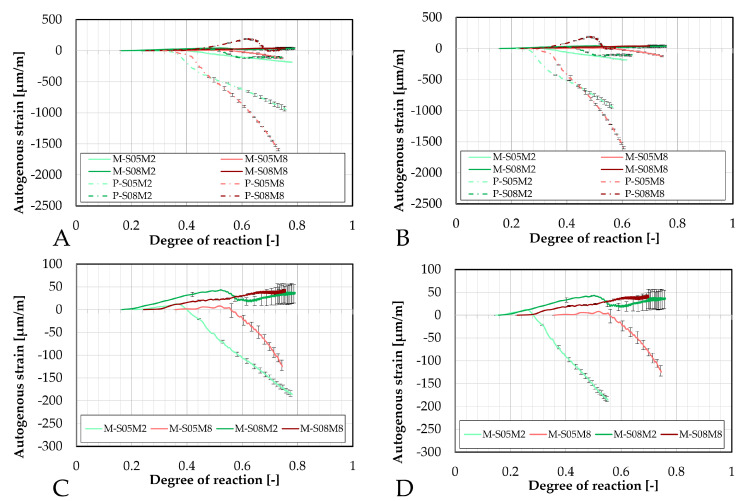
Comparison of the autogenous strain obtained with the corrugated tubes method for all AAS mortar compositions as function of the degree of reaction: (**A**). determined with polynomially fitted ultimate heat compared to the paste samples, (**B**). determined with the exponentially fitted ultimate heat compared to the paste samples, (**C**). determined with polynomially fitted ultimate heat, (**D**). determined with the exponentially fitted ultimate heat.

These results have also been compared in terms of the degree of reaction, as shown in Figure 6. For the S/B = 0.5 compositions, similar to previous observations, the addition of sand substantially reduces autogenous shrinkage. Furthermore, the onset of shrinkage is delayed in terms of the degree of reaction. Notably, M-S05M8 exhibits a later onset of shrinkage compared to both its corresponding paste scales and to M-S05M2. However, this delayed shrinkage is associated with a steeper slope in the strain curve.

A delayed shrinkage onset is generally beneficial when considered in terms of DOR, as it indicates that the reaction is more advanced and the material possesses higher tensile strength to better resist the restrained shrinkage in externally restrained conditions.

For the higher S/B compositions, the behavior differs, similarly to the results as a function of equivalent age. Specifically, M-S08M2 shows early-age swelling followed by significantly reduced shrinkage relative to the paste scale. After the shrinkage appears to stabilize, a little bit of swelling reoccurs. Again, the addition of sand delays the onset of shrinkage, thereby extending the swelling phase.

In contrast, M-S08M8 exhibits less swelling than its corresponding paste scale composition, with an accelerated onset of the swelling. Despite the reduced swelling, no shrinkage is observed throughout the duration of the test, while significant shrinkage (relative to the swelling) was present in the paste. Instead, as the DOR progresses, swelling continues to increase, eventually surpassing the apparent swelling M-S08M2 at DOR_1_ (A) = 0.57 and DOR_2_ (B) = 0.55.

The magnitudes of the shrinkage and the swelling have been quantified and are reported in Table 6. These results are compared to the corresponding paste results discussed in Lacante et al. [20].

The swelling values presented in Table 6 correspond to the early-age swelling phase, which is very important to note as the 0.8 S/B compositions also exhibit swelling later on. The shrinkage values were calculated as the difference between the maximum swelling (reported in the table) and the strain measured at 300 h.

The inclusion of sand into the mix significantly reduced shrinkage by 74%, 82%, 94%, and 100% for S05M2, S05M8, S08M2, and S08M8, respectively. The results indicate that a higher activator concentration and a higher S/B ratio lead to a higher reduction of the shrinkage. However, since these percentages are based on the strain observed at 300 h, they may not fully represent the shrinkage behavior of these compositions and their reduction with respect to the paste scale. In fact, as mentioned just before, the S/B composition exhibits later age swelling. Therefore, an alternative reduction percentage was calculated based on the maximum shrinkage occurring after the maximum swelling. Using this method, the shrinkage reduction for S08M2 is 82%. The previous conclusions are still valid.

In addition, the impact of sand on apparent swelling was assessed. For S08M2, the apparent swelling increased by 19% as a result of the sand addition. Conversely, S08M8 exhibited an 80% reduction in swelling.

Before transitioning to the larger-scale tests using the BTJade setup, an important issue arose that must be addressed.

During the testing using the corrugated tubes method, a first type of river sand, referred to in the following part as Sand 1, was used. Unfortunately, at the beginning of the larger-scale testing campaign, it was discovered that the available quantity of Sand 1 had been considerably reduced, as it had also been used in other student projects. A replacement was found: a similar 0/4 river sand with comparable properties, hereafter referred to as Sand 2, obtained from CRIC-OCCN. The only notable difference between the two sands lies in their absorption coefficients, with Sand 2 exhibiting a slightly higher value. Given that the sand acts as an inert filler in the mixture, it was initially expected that this variation would not significantly influence the results.

**Figure 7:**
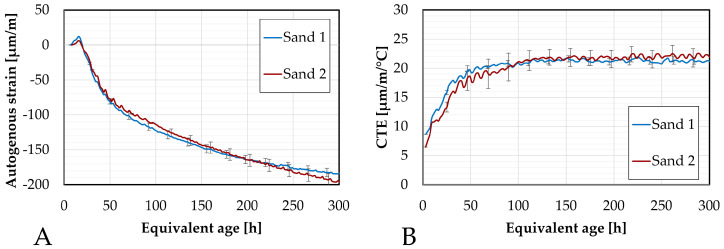
Differences between the two sands in terms of: (**A**). autogenous strain and of (**B**). CTE.

To assess and evaluate whether the change in sand type might introduce a discrepancy in the study, the autogenous strain as well as the coefficient of thermal expansion were investigated and compared for one selected composition (M-S05M2) using both Sand 1 and Sand 2. The comparison is presented in Figure 7.

Minor differences can be observed between the results obtained with Sand 1 and Sand 2, particularly in the amount of swelling at the beginning of the test, around 100 h, and towards the end of the test.

Two aspects were analyzed to assess these differences. First, the relative percentage difference in strain has been computed at selected ages. Second, the maximum standard deviation was also calculated. This was done for both the autogenous strain and the coefficient of thermal expansion. These values are summarized in Table 7.

The highest difference in autogenous shrinkage (37%) was observed at earlier ages due to the higher strain rate during that period. This decreases over time, which explains the more significant discrepancies at early ages compared to later ages. A maximum difference of 16% was found for the coefficient of thermal expansion.

The standard deviations were also evaluated for both parameters: yielding 7.5 µm/m for the autogenous strain and 1.8 µm/m/°C for the CTE. The standard deviation for the autogenous strain is within acceptable limits according to the ASTM C1698-09 standard [54], which prescribes a standard deviation (used as repeatability criterion) of 28 µm/m for mortar samples. No specific criterion could be found for the CTE. However, it is worth noting that despite the relatively high percentage error, the absolute standard deviations remain relatively small. Moreover, as seen in Figure 7, even though the curves do not perfectly overlap, the results for both sands generally fall within the error margins of the other.

#### 4.4.2. BTJade

The second method used to assess the autogenous strain is the BTJade device. It is based on the same principle as the corrugated tube method but is applied to larger scale samples.

**Figure 8:**
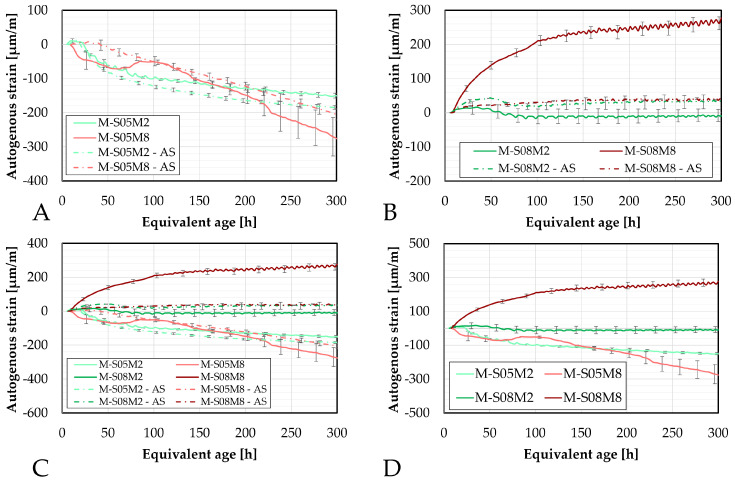
Autogenous strain of AAS mortars obtained with the BTJade device, presented as function of the equivalent age: (**A**). Compositions activated with S/B = 0.5 (compared to the equivalent corrugated tubes method), (**B**). Compositions activated with S/B = 0.8 (compared to the equivalent corrugated tubes method), (**C**). Comparison of all mortar compositions, (**D**). Comparison of the mortar compositions tested with BTJade. — = BTJade results, - · - : Corrugated tubes method.

Figure 8 presents the autogenous strain results obtained using the BTJade device, compared to the results obtained with the corrugated tubes. The standard deviation between the two methods for the swelling is 1.16, 5.40, 19.30 and 186.78 µm/m for M-S05M2, M-S05M8, M-S08M2 and M-S08M8, respectively, while for the shrinkage at 240 h, it is 21.83, 44.93, 48.22, 334.03 µm/m for M-S05M2, M-S05M8, M-S08M2 and M-S08M8, respectively. In general, the 2 M compositions yield better agreement between the two testing methods. For M-S08M2, the swelling is less pronounced with the BTJade method compared to the corrugated tube method. However, this was expected based on previous observations with PC-based materials [21,55]. Overall, these compositions exhibit the same behavior and yield similar results across both methods.

On the other hand, the results for the 8 M compositions are more complex. In the corrugated tube tests, some early-age swelling was observed. In contrast, with the BTJade method, shrinkage appears to be partially delayed, with swelling becoming visible around 75 h. Shrinkage resumes after approximately 110 h. After 225 h, both methods exhibit similar behaviors.

For M-S08M8, the swelling observed in the corrugated tube method was significantly lower than that on the paste scale. However, with the BTJade method, significantly magnified swelling is observed. This is comparable to the magnitude seen in the paste scale and reaches similar strain values around 100 h.

**Figure 9:**
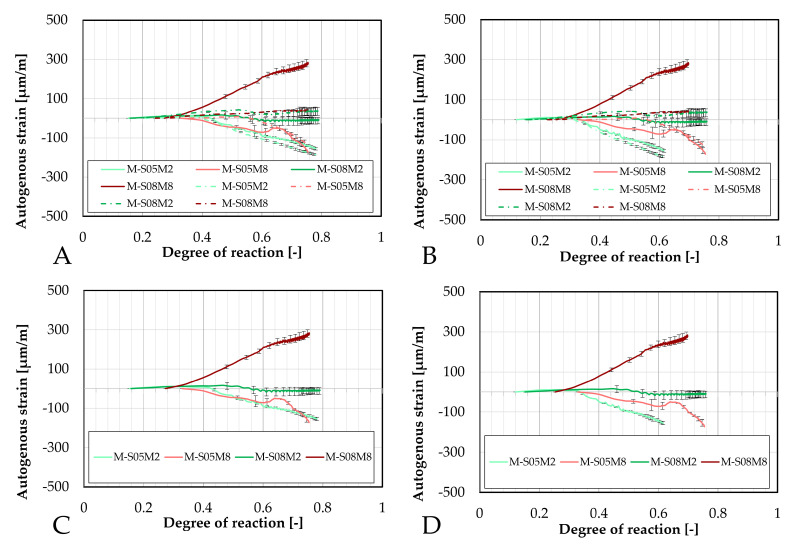
Comparison of the autogenous strain obtained with the BTJade device for all AAS mortar compositions as function of the degree of reaction: (**A**). determined with polynomially fitted ultimate heat compared to corrugated tubes method results, (**B**). determined with the exponentially fitted ultimate heat compared to corrugated tubes method results, (**C**). determined with polynomially fitted ultimate heat, (**D**). determined with the exponentially fitted ultimate heat. — = BTJade results, - · - : Corrugated tubes method.

The autogenous strain of AAS mortar monitored with the BTJade are presented in Figure 9 as a function of the degree of reaction. In terms of DOR, the autogenous strain for M-S05M2 appears to be slightly delayed. In contrast, M-S05M8 exhibits a notably late onset of its swelling, after which the behavior between the two testing methods becomes very similar.

For P-S05M8, the setting time is very fast. Given that this test requires a non-negligible amount of time to prepare and set up, it is possible that the setting time of the material was delayed in the testing process.

Even though the swelling of M-S08M2 is less pronounced in the BTJade method compared to the corrugated tubes method, both methods exhibit similar behavior with respect to the DOR (as well as the age) which is consistent with earlier observations.

As expected, the autogenous strain of M-S08M8 differs significantly between these two testing methods over time.

Furthermore, the different methods used to determine the DOR do not appear to influence the observed differences between M-S08M2 and M-S08M8.

### 4.5. Later-Age Autogenous Strain

The later-age autogenous strain was also investigated at the mortar scale, motivated by the significant findings observed at the paste scale. Two distinct groups emerged linked to the two different molar concentrations used.

Similarly to the early-age tests, two different sizes of specimens have been monitored to relate to the two sizes investigated. The smaller ones are 25 mm × 25 mm × 28.5 mm specimens, while the bigger ones are 100 mm × 100 mm × 40 mm specimens. These will be referred to as the DEMEC samples, because they are measured with the DEMEC device.

First, the results will be compared with the continuous measurement methods introduced earlier to assess the accuracy of these methods at early ages. Following that, the later-age behavior will be investigated.

For consistency, the manual testing results were initialized using the strain measured at that same age with the continuous measurements to ensure the most accurate comparison possible.

#### 4.5.1. Comparison at Early-Age

**Figure 10:**
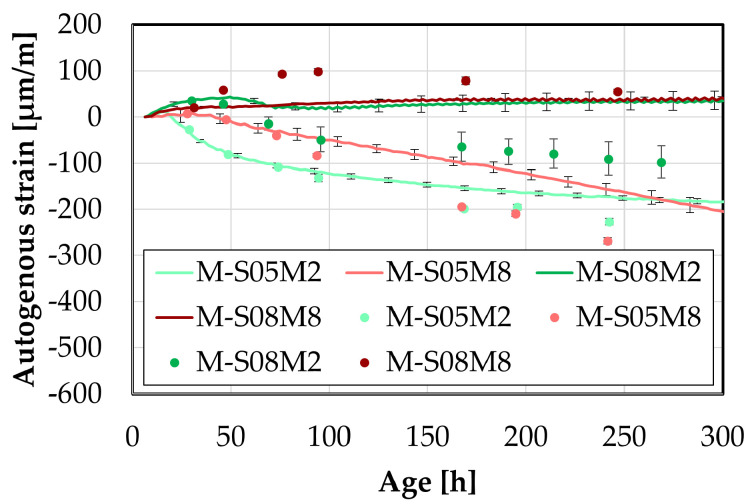
Early-age comparison between small DEMEC results and corrugated tubes method. Line = corrugated tubes method, dots = DEMEC results.

First, the results from the small manual samples are compared to those obtained with the corrugated tubes method, see Figure 10. During the first 75 h, there is good overlap between both methods for M-S05M2 and M-S05M8. Conversely, the swelling of M-S08M2 is mostly missed in the manual test, whereas the swelling observed in M-S08M8 is more apparent in the manual measurements. The standard deviation between the two methods for the swelling is 8.65, 5.40, 30.76, 109.94 µm/m for M-S05M2, M-S05M8, M-S08M2 and M-S08M8, respectively, while for the shrinkage at 240 h, it is 42.78, 127.74, 134.95, 193.73 µm/m for M-S05M2, M-S05M8, M-S08M2 and M-S08M8, respectively.

Overall, the behavior for S/B = 0.5 is quite similar between the two methods. Conversely, for the S/B = 0.8 compositions, the manual testing records more shrinkage.

Since the tests are conducted differently, some discrepancies between results is expected. Nevertheless, qualitatively, the behavior observed is similar across both methods.

**Figure 11:**
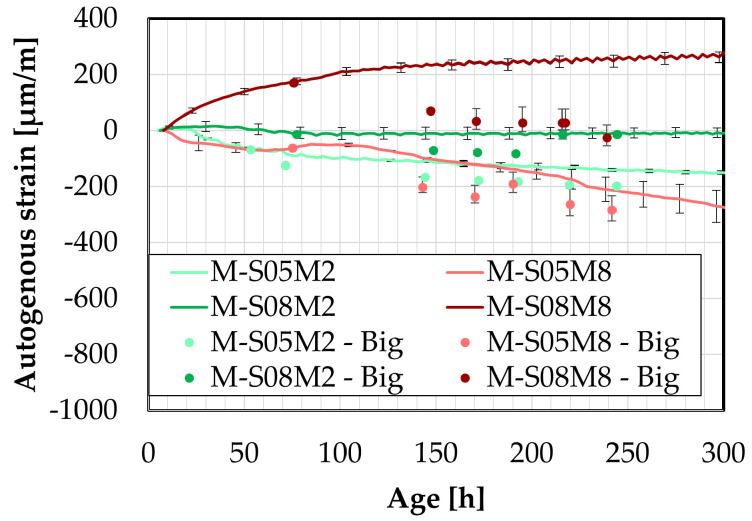
Early-age comparison between big DEMEC results and BTJade method. – = corrugated tubes method, · = DEMEC results.

Regarding the big samples, the comparison yields similar results to those observed with the smaller samples, see Figure 11. The standard deviation between the two methods for the swelling is 7.48, 0.00, 11.47, 218.88 µm/m for M-S05M2, M-S05M8, M-S08M2 and M-S08M8, respectively, while for the shrinkage at 240 h, it is 48.15, 49.73, 63.63, 411.54 µm/m for M-S05M2, M-S05M8, M-S08M2 and M-S08M8, respectively. At S/B = 0.5, there is a good correlation between the two methods, even better than for the smaller samples. For M-S08M2, the correlation between methods seems better, although some shrinkage is detected at certain points in the DEMEC results.

Finally, for M-S08M8, the two methods yield different results: shrinkage is measured with the manual method, whereas only swelling was recorded with the continuous method.

**Figure 12:**
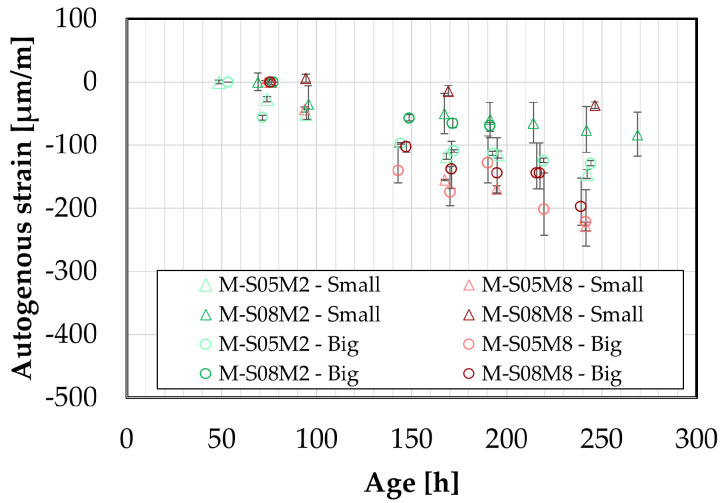
Early-age comparison between small and big DEMEC results.

Lastly, a comparison between the two types of DEMEC samples is presented in Figure 12, with results for each composition initialized at approximately the same time. The standard deviation between the two methods for the swelling is 0, 0, 0, 142.04 µm/m for M-S05M2, M-S05M8, M-S08M2 and M-S08M8, respectively, while for the shrinkage at 240 h, it is 16.46, 33.08, 23.10, 271.24 µm/m for M-S05M2, M-S05M8, M-S08M2 and M-S08M8, respectively. Except for M-S08M8, a good correlation between the two sample sizes is observed. For M-S08M8, however, the shrinkage measured on the smaller samples is noticeably lower than that on the larger samples.

#### 4.5.2. Later-Age Results

**Figure 13:**
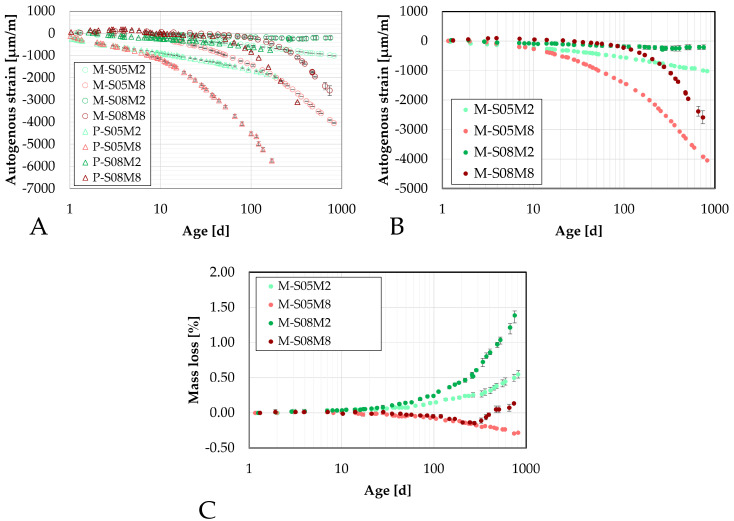
Autogenous strain of AAS mortar measured on the small DEMEC samples as function of the age: (**A**). comparison with the paste scale, (**B**). Comparison of the mortar scale, (**C**). Mass loss evolution.

The later-age DEMEC results are presented in this section. First, the results on the small DEMEC samples are analyzed and compared to the paste samples. Similar to the early-age results, a clear reduction in the magnitude of the autogenous shrinkage is observed with the addition of sand to the paste, see Figure 13A. As with the paste scale, two distinct groups emerge from the later-age results, based on the molar concentration. On the mortar scale, M-S08M8 exhibits a higher shrinkage slope compared to M-S05M8, whereas the opposite trend is observed for the 2 M compositions. The mass loss of each composition has been assessed on the three samples and is reported in Figure 13C. Compositions M-S08M2 exhibits a relatively higher mass compared to the other compositions, reaching 1.39% at 750 days of age. At the age of 338 days, an extra layer of aluminum was added to one of the samples to attempt reducing the mass loss rate which was by then 0.79% on that sample. This did not alter the tendency. All samples follow the same trend. In addition, no mass loss limit is imposed by the followed standards. Therefore, the results are considered despite the high mass loss. Finally, a high mass loss would be related to drying. Sirotti et al. [66] investigated the drying shrinkage of these compositions. They reported a mass loss of at least 5% at 260 days of age (depending on the relative humidity of the drying conditions) for a drying shrinkage of about 2000 µm/m at 75% relative humidity, while this mass loss was 0.55% in sealed conditions around the same time for an autogenous shrinkage of 265 µm/m. Moreover, two extra compositions had been included in the testing campaign but have not been presented in this paper: M-S05M05 and M-S08M05. These are AAS mortars made with a 0.5 M NaOH activator and a S/B = 0.5 and 0.8, respectively. Both these compositions exhibited a high mass loss as well: 0.78% at 730 days of age and 1.83% at 750 days of age, respectively. This indicates that the mass loss is closely linked to the concentration and the S/B ratio. Finally, the deviation of one of the M-S08M8 samples was observed thanks to the mass loss. Consequently, this sample’s data was excluded from the results.

**Figure 14:**
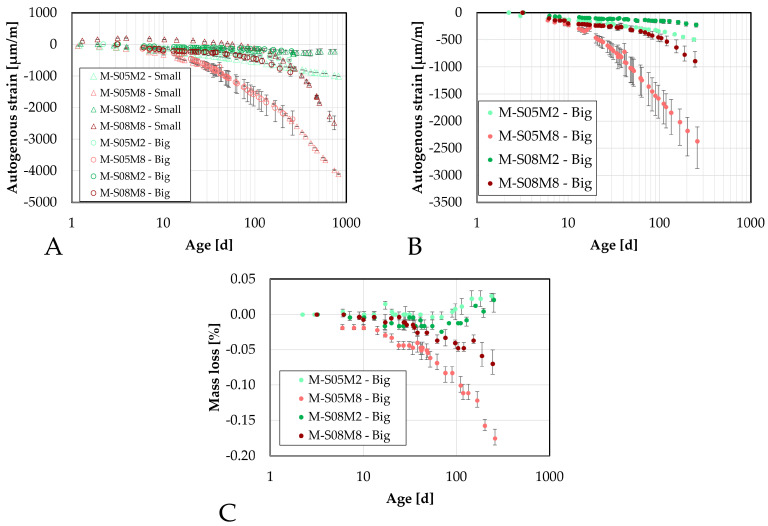
Autogenous strain of AAS mortar measured on the big DEMEC samples as function of the age: (**A**). comparison with the small scale, (**B**). Comparison of the mortar scale, (**C**). mass loss evolution.

The later-age results for the big samples are presented in Figure 14. These results are first compared with those from the small DEMEC samples. A good agreement is observed between the two sets of results for M-S05M2, M-S08M2, and especially M-S05M8. Although there is an initial discrepancy for M-S08M8 during the first few days, both sets start following similar trends over time.

While M-S08M8 does not exhibit significant shrinkage at the start of the test, its shrinkage rate increases over time. In contrast, the shrinkage slope for M-S05M8 becomes linear with respect to the logarithmic scale and remains relatively constant throughout the test duration.

In conclusion, a good agreement is observed across the different testing methods for composition M-S05M2. The consistency remains satisfactory for M-S05M8 and M-S08M2, with only minor discrepancies. Conversely, composition M-S08M8 shows significant variability depending on the geometry of the sample and the type of test. In some cases, high shrinkage is measured, while in others, high swelling is measured.

### 4.6. Coefficient of Thermal Expansion

Similarly to the early-age autogenous strain tests, the early-age evolution of the coefficient of thermal expansion was monitored using two different devices: the corrugated tube method and the BTJade device. Both of these methods were adapted to simultaneously monitor the evolution of the autogenous and thermal strains of the material.

#### 4.6.1. Corrugated Tubes Method

**Figure 15:**
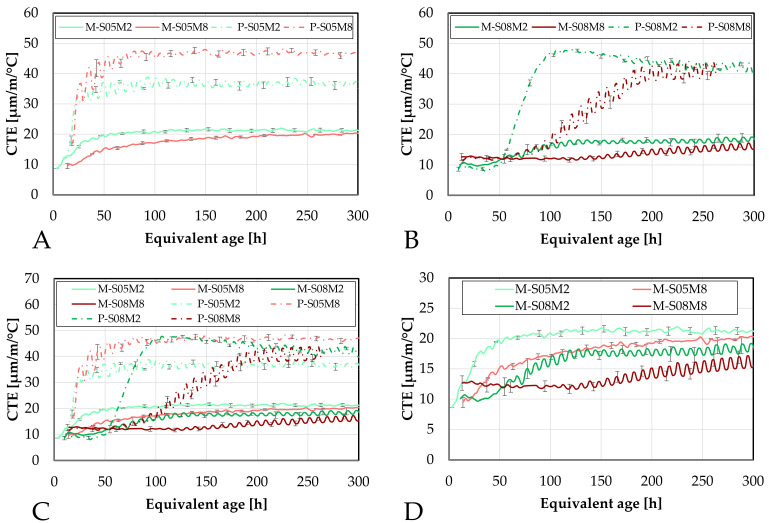
Coefficient of thermal expansion of AAS mortars obtained with the corrugated tubes method, presented as function of the equivalent age: (**A**). Compositions activated with S/B = 0.5 (compared to the equivalent paste compositions), (**B**). Compositions activated with S/B = 0.8, (**C**). Comparison of all mortar and paste compositions, (**D**). Comparison of the mortar compositions. M = mortar, P = paste.

First, the evolution of the CTE is investigated using the corrugated tube method and compared to the results obtained on the paste scale, see Figure 15.

The addition of sand significantly reduces the coefficient of thermal expansion, as expected. This is related to the lower CTE of sand and aggregates in general. When introduced into the material, this lowers the overall CTE compared to that of the paste alone [67].

Interestingly, M-S05M8 exhibits a slightly lower CTE than M-S05M2, while this was not observed on the paste scale. This might be related to the aggregate content *V_a_* in each mortar composition. Specifically, M-S05M8 has a *V_a_* of 0.45, which is slightly higher than that of M-S05M2 (*V_a_* = 0.42). This is because this factor depends on the density of each paste. Because both mortars were made with the same paste-to-sand mass ratio, a higher paste density results in a higher volume of sand in the mix. Therefore, it reduces the CTE a bit more.

Another notable observation is that M-S08M2 yields a lower CTE than M-S05M2 after the sand addition. The opposite was observed for the paste scale. In this case, the reduction cannot be attributed to aggregate content, as the 0.8 S/B compositions have a systematically lower *V_a_* than other corresponding 0.5 S/B compositions. Unexpectedly, M-S05M2 exhibits the highest CTE among the mortars, despite having the lowest at the paste scale. Additionally, the small and slow peak previously observed on the CTE of P-S08M2, after wich the CTE decreased, is not present anymore with the inclusion of sand.

Finally, the minimum CTE value of M-S08M8 is higher than that of the other mortar compositions, consistent with observations on the paste scale. This indicates that the S08M8 compositions have a more thermally reactive matrix skeleton than the other compositions.

**Figure 16:**
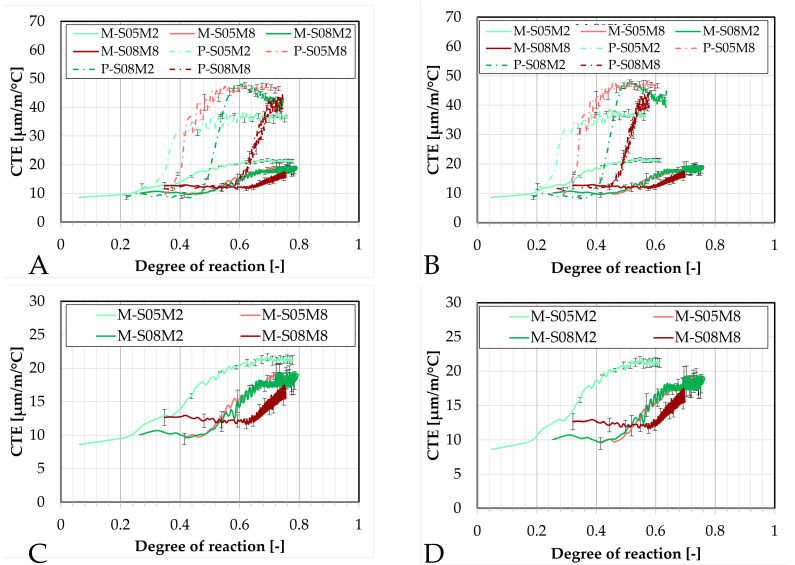
Comparison of the coefficient of thermal expansion obtained with the corrugated tubes method for all AAS mortar compositions as function of the degree of reaction: (**A**). determined with polynomially fitted ultimate heat compared to paste results, (**B**). determined with the exponentially fitted ultimate heat compared to the paste results, (**C**). determined with polynomially fitted ultimate heat, (**D**). determined with the exponentially fitted ultimate heat. — = mortar results, - · - : paste results.

The evolution of the CTE can be found as a function of the DOR (computed both ways) on Figure 16.

Concerning the 0.5 S/B compositions, the onset of the CTE evolution appears to be delayed compared to the corresponding paste compositions. As discussed previously, this behavior depends on how the ultimate heat is defined. In particular, for M-S05M8, both methods yield nearly identical values for the ultimate heat, resulting in very similar curves in Figure 16. In contrast, the other compositions exhibit a faster evolution in terms of DOR. Overall, the 8 M compositions show a delayed evolution with respect to the DOR when compared to their corresponding 2 M compositions.

**Figure 17:**
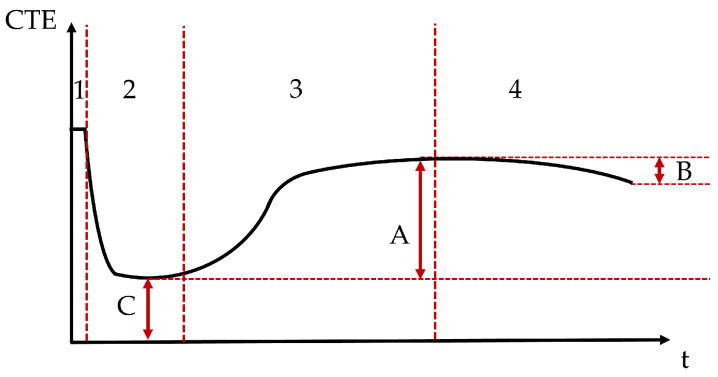
Parameters characterizing the coefficient of thermal expansion [71].

The coefficient of thermal expansion of such materials can be characterized by 4 stages [55] and by three parameters, see Figure 17. Stage 1 corresponds to an initially high CTE. This high value is related to the dominant influence of the solution present in the mix [68]. As the setting has not occurred yet, there is no solid skeleton in the mix. Therefore, the CTE is high because the CTE of liquids is higher than that of the solids [68]. This first stage is usually missed during the testing of the compositions in the present paper because it occurs too rapidly to be monitored correctly or to be seen at all. The initial setting of the material corresponds to the age at which the CTE is not that of the liquid anymore [57]. As setting happens, the evolution of the CTE enters stage 2. During that time, the CTE decreases fast and strongly because the free water gets consumed (“pre-setting”) [68]. In stage 2, Parameter C is introduced and corresponds the CTE of the matrix skeleton [55]. The lower the W/C ratio is, the faster the minimum value of CTE occurs after the setting time [68]. Then, stage 3 starts where the CTE is increased by parameter A. Depending on the water-to-cement (W/C) ratio, Parameter A is either equal to 0 (concrete with W/C = 0.74 [68]) or positive (concrete or mortar with W/C < 0.65 [21,57,58,68]). The increase has been explained by the internal drying that progresses due to the self-desiccation and the decrease in internal relative humidity [69,70]. In the case of the high W/C, the CTE does not vary after obtaining the minimum CTE value because the water content remains high during the reaction [68]. Finally, stage 4 is the long term CTE evolution and can be related to Parameter B which which characterizes the longer-term decrease in CTE over time [21,57]. This parameter can also be 0 [55,58,68].

**Figure 18:**
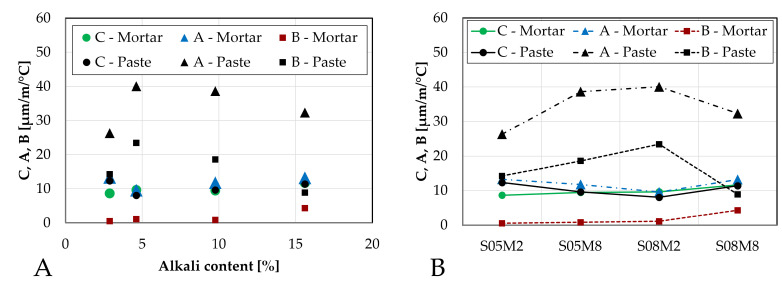
Parameters C, A and B characterizing the CTE of AAS mortars compared to the pastes: (**A**). as function of the alkali content; (**B**). as function of the compositions.

Figure 18 compares these parameters for the mortar compositions with those of the corresponding paste compositions determined in Lacante et al. [20]. Parameters A and B are significantly influenced by the addition of sand. Parameter A, describing the early-age increase of the CTE, is reduced and its trend is the opposite of that on the paste scale. In the mortar compositions, parameter A first decreases with increasing alkali content and begins to increase after about 5% of alkali content is obtained. Additionally, similarly to PC-based concretes [21,68], parameter A increases when the S/B decreases. However, this is not always the case for PC-based mortars [21]. Delsaute [21] obtained an CTE increase between 2.6 (PC-based mortar with W/C = 0.4) and 6.2 µm/m/°C (PC-based mortar with BFS and limestone filler with W/B = 0.4) for PC-based mortar with W/C between 0.3 and 0.6. These are similar values as for the AAS mortar results. This is relatively lower than parameter A obtained for AAS mortars. This also explains the lower CTE values (<16 µm/m/°C) for PC-based mortars during stage 3 compared to the AAS mortars.

Parameter B, representing the long term decrease in CTE, is nearly zero for all compositions except for M-S08M8. The low values of parameter B might be due to the assumption that the final measured CTE value in the test is assumed to be the long-term CTE, which is in reality not the case. The latter is related to the fact that M-S08M8 has the highest variability over time.

Interestingly, parameter C, which is related to the CTE of the matrix skeleton, is only lightly affected by the presence of sand. Only M-S05M2 shows a slight reduction, while the other mortar compositions remain close to their corresponding paste compositions, for parameter C. This suggests that the sand has a CTE similar to that of the paste skeleton. The mean value of parameter C for the mortar compositions is 9.86 (±1.10) µm/m/°C while it was 10.35 (±1.64) µm/m/°C for the paste compositions. Delsaute [21] reported a minimum CTE value between 9 (PC-based mortar with BFS and limestone filler with W/B = 0.4) and 12 µm/m/°C (PC-based mortar with W/C = 0.6) for PC-based mortar with W/C between 0.3 and 0.6. These are similar values to the AAS mortar results.

Finally, a relation between the evolution of the autogenous strain and the evolution of the CTE can be observed, especially in terms of degree of reaction. It seems that the CTE and autogenous shrinkage start to increase around the same DOR. This had also been observed by Delsaute an Staquet [57] on PC-based concrete. Similarly, this occurs around DOR = 0.5 for M-S05M8 and M-S08M2.

#### 4.6.2. BTJade

**Figure 19:**
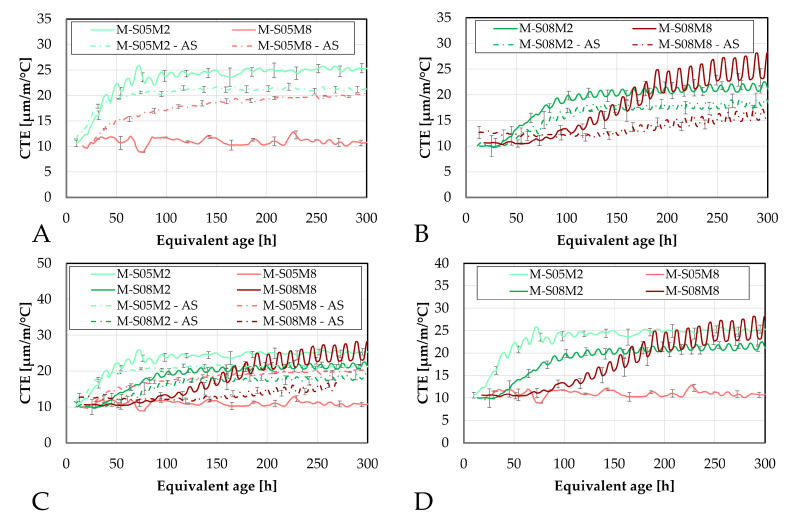
Coefficient of thermal expansion of AAS mortars obtained with the BTJade method, presented as function of the equivalent age: (**A**). Compositions activated with S/B = 0.5 (compared to the corrugated tubes method), (**B**). Compositions activated with S/B = 0.8, (**C**). Comparison of all mortar compositions on both methods, (**D**). Comparison of the mortar compositions with BTJade. — = BTJade, -·- = Corrugated tubes.

In the present section, the coefficient of thermal expansion will be investigated using the BTJade device and compared to the results obtained with the corrugated tubes method, see Figure 19.

Similarly to the autogenous strain results, the M-S05M2 and M-S08M2 compositions yield the most similar results across both methods. Conversely, the 8 M compositions exhibit more differences. Interestingly, M-S05M8 yields a significantly lower CTE when measured with the BTJade device, staying close to the CTE of the matrix skeleton, despite maintaining a satisfactory error.

On the other hand, M-S08M8 shows a considerably higher CTE with the BTJade method, even surpassing that of the M-S08M2 composition. However, the same unusual behavior is observed once again. The onset of CTE evolution for M-S08M8 is consistently delayed and increases slowly. Overall, the BTJade method tends to yield higher CTE values than the corrugated tubes method. Interestingly, in this case, M-S08M8 does not exhibit the initially elevated CTE values that were observed in other S08M8 compositions.

Finally, the error between both methods was assessed. For M-S05M2 and M-S08M2, average errors of 16% (maximum 21%) and 19% (maximum 24%) were found, respectively; with corresponding average standard deviations of 2.33 µm/m/°C (maximum 3.13 µm/m/°C) and 2.00 µm/m/°C (maximum 2.93 µm/m/°C), respectively. For M-S05M8 and M-S08M8, average errors of 37% (maximum 47%) and 31% (maximum 82%) were found, respectively; corresponding to standard deviations of 4.86 µm/m/°C (maximum 6.74 µm/m/°C) and 3.84 µm/m/°C (maximum 8.96 µm/m/°C), respectively.

**Figure 20:**
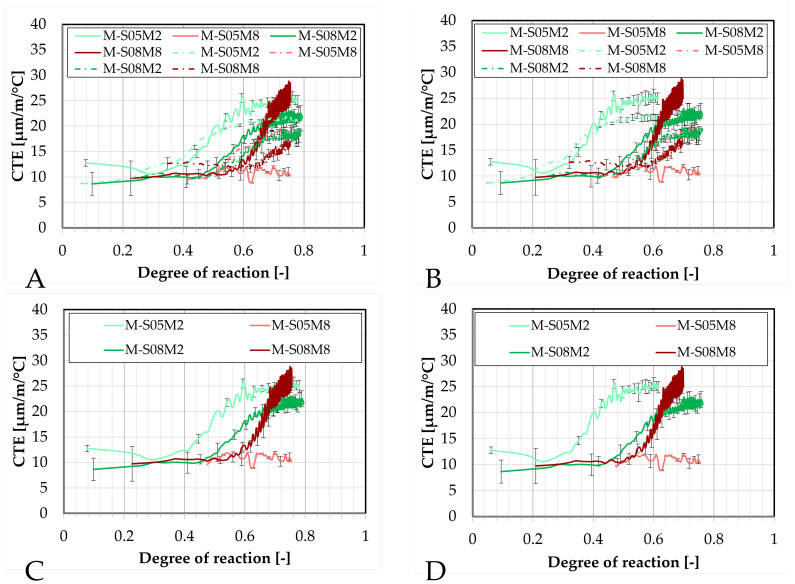
Comparison of the coefficient of thermal expansion obtained with the BTJade method for all AAS mortar compositions as function of the degree of reaction: (**A**). determined with polynomially fitted ultimate heat compared to the corrugated method, (**B**). determined with the exponentially fitted ultimate heat compared to the corrugated method, (**C**). determined with polynomially fitted ultimate heat, (**D**). determined with the exponentially fitted ultimate heat. (— = BTJade results, - · - = Corrugated tubes method).

The coefficient of thermal expansion is presented as a function of the degree of reaction (computed both ways) in Figure 20.

For M-S05M2, the CTE measured with the corrugated tubes method appears to increase slightly earlier, whether it is considered in terms of equivalent age or degree of reaction. In contrast, for M-S08M2 and M-S08M8, the CTE evolution is more delayed in the corrugated tubes method compared to the BTJade device. Once again, the most notable observations are the significantly low CTE measured for M-S05M8 and the high CTE for M-S08M8 when using the BTJade method.

Concerning M-S05M8, the issue may be primarily related to the rapid loss of workability. The combination of higher alkali concentration and lower S/B ratio leads to a fast-setting paste with reduced workability and accelerated workability loss [72]. Initial slump measurements confirmed this. The standard test yielded a very low diameter and the “hit” slump also yielded quite low values.

Consequently, this larger-scale BTJade test setup might not be ideal for this composition. The preparation and assembly of the BTJade specimens takes a non-negligible amount of time, and the larger sample size compared to the corrugated tubes leads to more rapid temperature rise, further accelerating workability loss. Moreover, completing the BTJade setup requires placing the head component on top of the head fixed to the material. This may involve minor movement or adjustments of the specimen, which can impact the fast-setting materials. In contrast, the corrugated tubes method offers more flexibility in handling and does not require any further manipulation of the specimen after the casting. Therefore, the corrugated tubes method might be more suitable for testing materials with such a rapid setting behavior.

As demonstrated in previous sections, the addition of sand significantly reduces both the autogenous strain and the coefficient of thermal expansion. This reduction is likely attributed, on one hand, to the increased proportion of inert material in the mixture [31], which is approximately 40 to 45% of (volume) aggregate content in these cases. Additionally, the presence of sand introduces local restraints, as the paste shrinks around the aggregate particles [22].

## 5. Results and Discussion of the Investigation of the Upscaling from Mortar to Concrete

### 5.1. Compressive Strength

In the preliminary campaign, the compressive strength was evaluated at three different ages: 1 day, 2 days and 7 days. For this purpose, one 10 cm × 10 cm × 10 cm cube was cast for each testing age. Subsequently, during the in-depth testing campaign, additional cubes were cast for the C-S05M2 and C-S08M8 compositions, in order to have two data points at the following ages: 1 day, 3 days, 7 days and 28 days. As a result, the compressive strength results presented for C-S05M2 and C-S08M8 are an average of two data points for all ages, except for the two-day age test, for which only one cube was tested. For C-S08M2, only one cube was tested per testing age. Even though different mixers were used (5 L Hobart mixer for the preliminary campaign and the 12 L mixer for the in-depth campaign), the error between results was within the margin given by the standards, indicating the negligible impact of using different mixers in terms of compressive strength.

**Figure 21:**
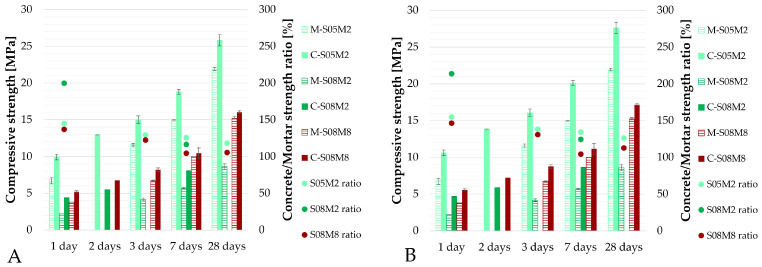
Compressive strength of: (**A**). AAS concrete cubes (10 × 10 × 10 cm^3^) compared mortar cubes (5 × 5 × 5 cm^3^), (**B**). AAS concrete cubes (equivalent 5 × 5 × 5 cm^3^) compared mortar cubes (5 × 5 × 5 cm^3^). Legend: bars: compressive strength, dots: compressive strength ratio between concrete and mortar scale.

Figure 21 compares the compressive strength of AAS concrete to that of AAS mortar. In Figure 21A, no correction is applied for the difference in specimen geometry, while in Figure 21B, the equivalence factor of 0.935 [21] is applied to account for the effect. The compressive strength results are in the same range as those obtained by Lee et al. [37], who investigated compositions with similar aggregate and sand content with respect to the amount of paste. Humad et al. [73] also reported similar 7-days compressive strengths. In contrast, Sofi et al. [74] obtained a higher compressive strength (41.8 MPa at 7 days and 56.5 at 28 days) as their mix formulations were designed to have a comparable compressive strength to the PC material’s 50 MPa. Li et al. [75], Bernal et al. [38] and Ma et al. [76] also reported higher compressive strength results for their AAS concrete compositions. The compositions also had sodium silicate as activator.

In this case, the compressive strength of AAS concrete is consistently higher than that of AAS mortar. However, the difference between the two reduces over time, nearing 0% at later ages. At 28 days, the ratio between the compressive strength of the concrete compositions and the mortar compositions (without applying the equivalence factor) is 118.0% and 105.4% for C-S05M2 and C-S08M8, respectively. When the equivalence factor is applied, the ratio increases to 126.2% and 112.7% for C-S05M2 and C-S08M8, respectively. When taking into account the equivalence factor, the compressive strength of concrete is increased. This increases the difference between the two scales. Between 7 days and 28 days, a non-negligent increase can be seen. This indicates that the reaction is still ongoing.

Klun et al. [63] reported increased compressive strength results on the concrete scale compared to the mortar scale up to 36 h, after which the opposite was observed. It remains challenging to find relevant data in the literature about the compressive strength of AAM, especially on the concrete scale and on the differences resulting from the upscaling, to be able to compare results. In general, compressive strength in both PC-based mortar and concrete is influenced by the aggregate content [77] and the water-to-cement ratio [21]. For AAM, this complexity [38] is increased by additional parameters such as the type of activator, its concentration, and the solution-to-binder ratio. All of which significantly affect strength development [20].

Based on the preliminary results, the compositions C-S05M2 and C-S08M8 were selected for further investigation. In fact, composition C-S08M2 still exhibited segregation in the specimens, decreasing the reliability of the results.

### 5.2. Splitting Tensile Strength

**Figure 22:**
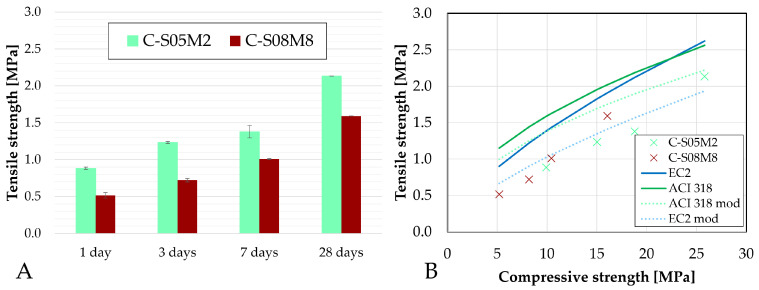
Tensile strength of AAS concrete: (**A**). as a function of the age, (**B**). as a function of the compressive strength (mod = modified prediction of the tensile strength).

The splitting tensile strength is the strength obtained directly from the tensile splitting test, also called the Brazilian test, as defined in EN 12390-6:2023 [78]. To estimate the actual tensile strength of the material, a reduction factor of 0.9 is applied to the splitting tensile strength, in accordance with the same standard [62]. The obtained tensile strength values are shown in Figure 22A as a function of the age.

Uppalapati [26] has evaluated the tensile strength of alkali-activated slag and fly ash based on the splitting tensile strength. Their results are almost twice as high as those obtained on the concrete scale presented here. In contrast, other studies by Lee et al. [37] and Sofi et al. [74] have reported splitting tensile strength of concrete in the same range as the results in the present section. However, Li et al. [75] reported only higher tensile strength results (ranging between 2.7 and 3.6 MPa) for alkali-activated slag and fly ash concrete. But in that case, the total mass of aggregates (coarse aggregate + sand) was lower, which could explain the increased tensile strength, similarly to the higher results on mortar scale.

The tensile strength is also compared to the compressive strength (without applying the equivalence factor). An almost linear relationship appears to exist between the two. The factor between the two is a very important parameter in order to be able to assess one when the other is known [79]. In addition, this allows to evaluate to which type of stress the concrete is more sensitive [75]. This factor can also be closely linked to the micro-cracking development in the material, especially in the paste that surrounds the aggregates. This is an interesting parameter since the tensile strength is more impacted by the micro-cracking than the compressive strength [80]. For C-S05M2, the compressive-to-tensile strength ratio 12.3 (±1.0), while for C-S08M8, it is 10.5 (±0.6) for C-S08M8. Li et al. reported a factor of 16.11 (±3.44), which is relatively higher than what is found in the present research. This discrepancy might be related to the material’s compositions (slag and fly ash activated by sodium hydroxide and sodium silicate, with a lower aggregate content). These results highlight the significant influence of the mix’s ingredients and quantities.

Several authors have mentioned the existence of a good correlation between the compressive strength and tensile strength for PC-based materials (such as described in the European standard EN 1992-1-1 Eurocode 2 [81] and the American standard ACI 318-08 [82]). Similar correlations have also been observed as well for AAM [26,37,39,74].

Equation (3) is proposed by Eurocode 2 while Equation (4) is proposed by the ACI 318.

(3) $$f_{ctm}=a\;\cdot \;f_{c}^{\frac{2}{3}}=0.30\;\cdot \;f_{c}^{\frac{2}{3}}$$

(4) $$f_{ctm}=0.9\;\cdot \;f_{ct}=0.9\;\cdot \;b\;\cdot \;f_{c}^{d}=0.9\;\cdot \;0.56\;\cdot \;f_{c}^{\frac{1}{2}}$$

Several authors have attempted to adapt the ACI equation in the following ways. Lee et al. [37] proposed a modified version of the ACI 318 equation, replacing factor *b* by 0.45, while Sofi et al. [74] found a better correlation for b=0.48, all while keeping b=0.5. On the other hand, Uppalapati [26] suggested b=0.52 and d=0.53 for mortar compositions.

Both equations have been modified in the present study, while keeping *d* constant for Equation (4). A good correlation was found for a=0.22 and 0.31. In general, the newly proposed factors are lower than those prescribed by the standards. The fitting can be found in Figure 22B (where “mod” denotes the modified prediction of the tensile strength). The RMSE was calculated for both the original equation and the adapted equation, with the values summarized in Table 8. The modification of the equations yielded better RMSE values, indicating more realistic tensile strengths.

### 5.3. Apparent Density

**Figure 23:**
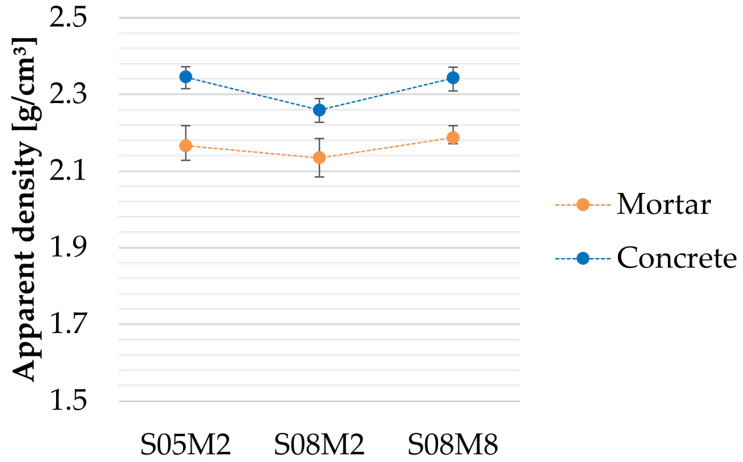
Apparent density of the AAS concrete compositions.

Next, the apparent density of the concrete was evaluated, see Figure 23. As expected, the density of the concrete compositions is higher than that of the mortar compositions [74], which in turn was higher than that of the paste compositions. This increase is due to the higher increase of the aggregate content. Indeed, coarse aggregates and sand have a higher density (2.65 g/cm^3^ and 2.62 g/cm^2^, respectively) than the binder (between 1.70 and 2.03 g/cm^3^). Since aggregates constitute about 70% by mass of the material, an overall increase in density is expected.

### 5.4. Early-Age Autogenous Strain

**Figure 24:**
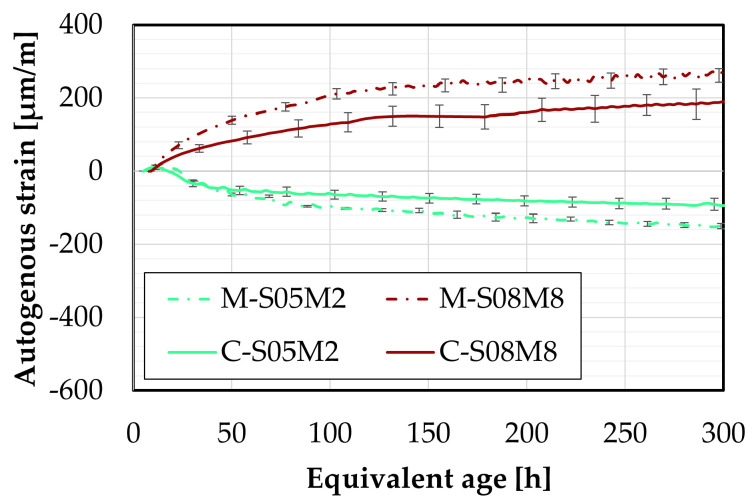
Autogenous strain of AAS concrete compositions compared to mortar compositions, as a function of the equivalent age.

**Figure 25:**
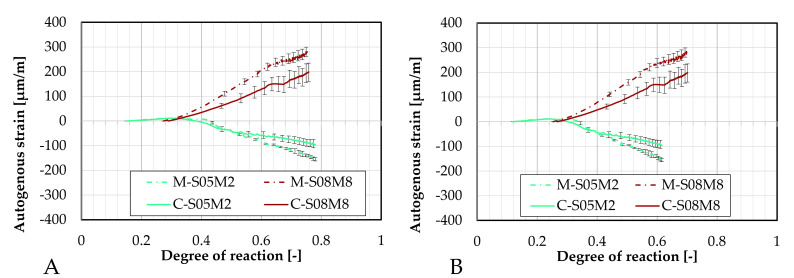
Autogenous strain of AAS concrete compositions compared to mortar compositions, as a function of the degree of reaction computed with the ultimate heat determined by: (**A**). the polynomial method, (**B**). the exponential method.

The autogenous strain of AAS concrete is compared to that of AAS mortars in Figure 24 and Figure 25, as a function of the equivalent age and the degree of reaction, respectively.

The swelling of C-S05M2 amounts to 11.2 µm/m which is only slightly higher than that of M-S05M2 (10.6 µm/m, corresponding to a 6.1% increase). On the other side, the addition of coarse aggregates to M-S05M2 yielded a reduction of the shrinkage from 162.1 µm/m (for M-S05M2) to 106.0 µm/m (for C-S05M2), leading to a reduction of 34.6% at 300 h.

For C-S08M8, no shrinkage was observed, similar to the mortar scale monitored with the BTJade method. Only swelling was visible. This measured swelling amounted to 189.4 µm/m, while it was 277.1 µm/m for the mortar scale (M-S08M8). This indicates that the addition of coarse aggregates to the M-S08M8 compositions led to a 31.7% decrease of swelling. This reduction is in the same range as the shrinkage reduction of C-S05M2. The reduction of the swelling had also been observed and discussed on the mortar scale. In fact, the global effect of the aggregates might hinder the swelling of the paste between the grains and the aggregates.

In general, the absolute value of the autogenous strain is reduced when upscaling from the mortar scale to the concrete scale, whether it is shrinkage or swelling. This trend is consistent with previous observations from paste to mortar scale, where a reduction in shrinkage and swelling was also noted during scale comparisons using the corrugated tube method.

In comparison with literature results, Li et al. [75] reported higher autogenous shrinkage (almost 300 µm/m) while the composition intended to mitigate the shrinkage reached nearly 100 µm/m. Humad et al. [73] reported higher autogenous shrinkage, with still a large increase until at least 10 days, depending on the composition. Ma et al. [76] yielded similar autogenous shrinkage at very early-age, but it increased much more later on. Conversely, Huang et al. [83] obtained relatively low autogenous shrinkage during the first day after which a high increase of the autogenous shrinkage could be seen. A constant (reduced) slope could be expected by 60 days of age for these materials (activated with sodium silicate) [40]. To summarize, the use of sodium silicate results in higher compressive strength but significantly increases the autogenous shrinkage. Finally, none of the researchers reported important swelling such as observed for C-S08M8. Only some very early-age swelling could be monitored in cases where it was tried to mitigate the shrinkage [75].

The autogenous shrinkage observed for C-S05M2 is slightly lower than the shrinkage that can be obtained for PC-based concretes, but this depends on the water-to-cement ratio and the type of cement that is used. Darquennes et al. [84] reported 80 µm/m for CEM I, 90 µm/m for CEM III/A and 135 for CEM III/B (W/C = 0.45 for all three compositions). Some swelling of about 90–95 µm/m was observed for the CEM III concretes. Delsaute et al. [57] obtained autogenous shrinkage of about 110 µm/m for concretes with W/C of 0.4 and 0.5 while the shrinkage was reduce to about 60 µm/m for a concrete with a W/C of 0.6 and 80 µm/m for a concrete with a W/C = 0.4 where part of the cement was replaced with BFS and limestone filler. They also reported swelling going up to 40 µm/m for concrete with W/C = 0.6, while about 170 µm/m was obtained for the concrete with W/C = 0.4 where part of the cement was substituted with BFS and limestone filler. Higher swelling was obtained with higher W/C for concretes with only cement. This is also the case for the AAS concretes.

The autogenous strains evolve relatively linearly with respect to the degree of reaction, which is not necessarily the case of PC-based concretes [57]. The swelling period is shorter for C-S05M2 compared to M-S05M2, similarly to PC-based materials [21]. Even though the shrinkage starts earlier in terms of DOR for C-S05M2 compared to M-S05M2, the shrinkage rate (slope) is less steep. As a result, the autogenous strain becomes lower from DOR_1_ = 0.50 and DOR_2_ = 0.38 onwards, where DOR_1_ and DOR_2_ are the degrees of reaction calculated with the ultimate heat determined with the polynomial and the exponential method, respectively.

On the other side, M-S08M8 and C-S08M8 also exhibit a relative linear behavior with in the early stages. Both compositions exhibit a decrease of slope of between DOR_1_ = 0.62 → 0.68 and DOR_2_ = 0.58 → 0.62. This decrease is less pronounced for M-S08M8. After this period, the slope increases again.

### 5.5. Early-Age Coefficient of Thermal Expansion

**Figure 26:**
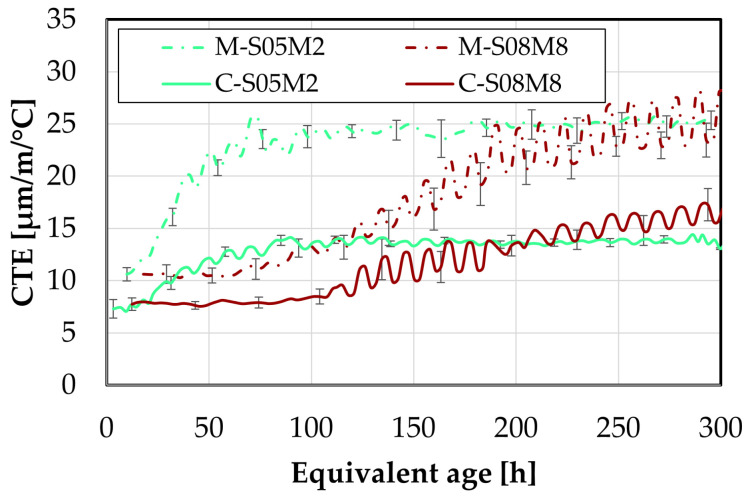
Coefficient of thermal expansion of AAS concrete compositions compared to mortar compositions, as a function of the equivalent age.

**Figure 27:**
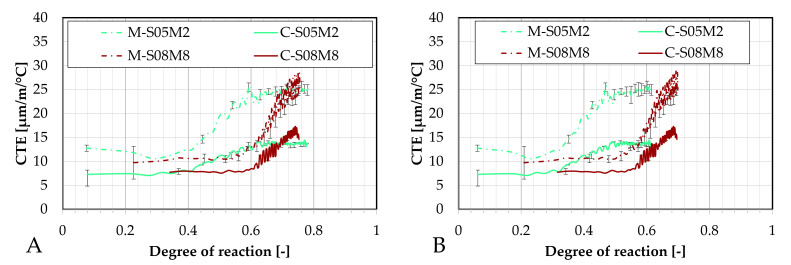
Coefficient of thermal expansion of AAS concrete compositions compared to mortar compositions, as a function of the degree of reaction computed with the ultimate heat determined by: (**A**). the polynomial method, (**B**). the exponential method.

Using the same test setup, the early-age development of the coefficient of thermal expansion of AAS concrete was monitored. The coefficient of thermal expansion of the AAS concrete compared to that of the AAS mortar compositions can be found in Figure 26 and Figure 27, as a function of the equivalent age and of the degree of reaction, respectively.

The addition of aggregates significantly reduces the coefficient of thermal expansion. The minimum CTE yields 7.39 µm/m/°C for C-S05M2 and 7.78 µm/m/°C for C-S08M8, while it was around 9.86 µm/m/°C for the mortar compositions. At 300 h, a CTE reduction of 48.0% is measured for the S05M2 composition while it was about 34.2% for the S08M8 compositions.

In the case of PC-based concrete, Delsaute and Staquet [57] reported a minimum value of the CTE between 7 and 10 µm/m/°C. After which, they observed an increase until 10 to 12 µm/m/°C for PC-based concrete and 14 µm/m/°C for a concrete where part of the cement is replaced by blast-furnace slag and limestone filler. The latter also exhibited the highest minimum CTE, indicating that the addition of BFS increases the CTE even in PC-based concrete. The CTE of AAS concrete seems to be slightly higher than the CTE of PC-based concrete. A lower thermal cracking risk can be expected in restrained conditions for concrete than for mortar and paste. This cracking risk due to restrained thermal shrinkage becomes comparable to the thermal cracking risk of PC-based materials as the scale goes from paste to mortar to concrete. It might even become smaller than that of PC-based concrete as the induced stress depends on the CTE and the E-modulus, which has been reported PC-based mortar similar or higher compared to AAS mortar [72]. Ma et al. [85] reported generally higher CTE results (initial CTE of 69 µm/m/°C, decreasing to 9–12 µm/m/°C and increasing again until at least 27 µm/m/°C at 65 h of age) for a low-calcium fly ash based concrete. However, their tests were conducted at elevated temperatures ranging from 27 °C to 63 °C. The curing temperature influences the coefficient of thermal expansion on the paste scale [71]. This effect might extend to the concrete scale, as suggested by this comparison. Zahabizadeh et al. [68] reported minimum CTE values of 11.8, 7.0 and about 9 µm/m/°C and for PC-based concretes with W/C = 0.38, 0.53 and 0.74, respectively. After this, the CTE increased until 14.2 and 7.7 µm/m/°C for the concretes with W/C = 0.38 and 0.53 while the last concrete did not increase for the rest of the test duration. It should be noted that these tests were stopped after maximum 100 h. Interestingly in the present paper, the CTE of C-S08M8 only starts increasing after about 100 h, before which it remained relatively constant.

In terms of degree of reaction, a closer look at the length of the initial stage shows that it does not appear to last longer on the concrete scale compared to the mortar scale. The CTE begins to increase around the same DOR on both scales. Consistent with observations on mortar, the CTE of C-S08M8 starts to increase later than that of C-S05M2. The previously mentioned relation between the autogenous shrinkage and the CTE can be observed for C-S05M2: both start to increase around the same DOR = 0.3, which is sooner than for PC-based concretes [57]. In addition, the CTE of C-S08M8 start to increase around DOR = 0.6, while a short decrease of the swelling slope could be observed on the autogenous strain results.

## 6. Conclusions and Perspectives

The following conclusions can be drawn from the mortar and concrete scale investigation.

- The compressive strength was slightly decreased with the inclusion of sand in the paste, to obtain mortar. The isothermal calorimetry results indicated that the sand was relatively inert. However, because of differences on the cumulative heat with respect to the one on paste, the ultimate heat was re-assessed and yielded results that were at most 26.8% reduced. This error depended on the extrapolation method.
- The autogenous strain and the coefficient of thermal expansion were investigated with different methods. Compositions like M-S05M2 yield very similar results, while compositions such as M-S08M8 did not produce similar results across different testing methods.
- The addition of the sand to paste reduced the autogenous shrinkage from 134–1195 µm/m to 0–217 µm/m, representing a reduction between 74 and 100%.
- The long-term autogenous strain was also investigated and it was reduced as well on the long term. Similarly to the paste scale, two groups emerged based on the concentration of the activator. Around 740 h of age, M-S05M2, M-S05M8, M-S08M2 and M-S08M8 exhibit an autogenous strain of −1024, −4005, −243 and −2606 µm/m respectively. In addition, M-S08M8 exhibits the highest shrinkage rate magnitude.
- The compressive strength of AAS concrete is higher than that of AAS mortar, yielding 8.1 (M-S08M2)–25.8 (M-S05M2) and 8.6 (C-S08M2)–21.9 (C-S05M2) MPa at 28 days, respectively. Nevertheless, the ratio of compressive strength of concrete over the compressive strength of mortar decreases over time.
- The tensile strength was evaluated based on the splitting tensile test. This yields a tensile strength (between 1.6 and 2.1 MPa) about 11 times lower than the compressive strength. A satisfactory equation was established based on standards. The one based on the EC 2 yielded a smaller error than that of the ACI 318.
- The autogenous strain, whether it was shrinkage or swelling, was once again reduced by adding coarse aggregates in the mortar mix, now yielding between −89.8 and 188 µm/m. The autogenous shrinkage was relatively lower than what can be found in the literature for similar materials. However, the results of C-S05M2 were in the same range as results found on PC-based materials. The addition of the sand has a higher restraining impact on the autogenous strain than the addition of coarse aggregates (when the sand is already there).
- The addition of coarse aggregates to mortar decreased the overall CTE, whether it is the minimum value (related to the CTE of the solid skeleton) or the final value. However, the addition did not delay the development. At 300 h, a CTE of 15 µm/m/°C can be expected for the AAS concrete, which is still slightly higher than the CTE of PC-based concrete. This implicates a lower thermal cracking risk in restrained conditions for concrete than for mortar and paste. It becomes comparable to that of PC-based materials as the studied scale goes from paste to concrete. This is because the volume fraction of the aggregates becomes higher.
- In general, it was seen that the comparison with the literature still remains quite difficult. Most concrete studies report results on slag and fly ash activated by sodium hydroxide and sodium silicate. In comparison, it seems that the addition of the sodium silicate activator strongly increases the compressive strength, and also the tensile strength sometimes. But it also strongly increases the autogenous shrinkage, even on the concrete scale.

The combination of the present study with investigation into the drying shrinkage contribution [66] to global volume changes could reveal interesting results, as this study is limited to sealed conditions. Another limitation of this research is the macroscale aspect. Therefore, the microstructural and pore solution evolution as well as the transport mechanisms and bound water content, should be studied on the mortar and concrete scale. This could help understand the effect of the sand and aggregates on the studied properties.

Further research should investigate the cracking activity of the material in free condition due to self-restraint, induced by the different aggregates added to upscale the paste. In fact, as the aggregates reduce the shrinkage by restraining the paste, resulting micro-cracking is possible. This should be investigated because it can affect the durability of the material. To fully assess the cracking risk due to thermal shrinkage in restrained conditions, the elastic modulus should also be investigated. Next, a testing campaign on the TSTM (temperature stress testing machine) combining shrinkage, CTE, creep and relaxation would increase the understanding of the early-age behavior of such materials. This would also contribute to the overall cracking risk assessment of AAS materials by determining if the reduced thermal part of the cracking risk compensates the potentially higher cracking risk due to restrained autogenous shrinkage and how the amplitude of the creep impacts this. A complete data set of AAS on the three scales has been created, comparing concentration and S/B effects as well as sand and coarse aggregates effects, making it possible to start modelling thermal and autogenous strains in AAS.

## Figures and Tables

**Table 1:** Chemical composition of the blast-furnace slag in mass percent from X-ray fluorescence spectroscopy.

Material	SiO_2_	Al_2_O_3_	Fe_2_O_3_	CaO	K_2_O	MgO	TiO_2_	SO_3_	Na_2_O	BaO	MnO
Slag	34.20	12.86	0.35	39.95	0.62	7.91	1.13	1.88	0.50	0.13	0.30

**Table 2:** Characteristics of the studied mortar compositions.

Composition	Alkaline Concentration [mol/L]	S/B [-]	W/B [-]	Alkali Content [%]	Paste/Sand [-]
M-S05M2	2	0.5	0.46	2.88	1/1
M-S05M8	8	0.5	0.37	9.76	1/1
M-S08M2	2	0.8	0.74	4.61	1/1
M-S08M8	8	0.8	0.60	15.61	1/1

**Table 3:** Compositions studied at the concrete scale.

Composition	Solution	Solution	Water	Slag	Sand	Aggregates	Aggregates	Aggregates
	Concentration [mol/L]	[g]	[g]	[g]	0/4 [g]	6/10 [g]	10/14 [g]	14/20 [g]
C-S05M2	2	811.4	746.5	1622.8	2434.2	1641.7	816.2	853.3
C-S08M2	2	1061.9	982.3	1327.4	2389.3	1641.7	816.2	853.3
C-S08M8	8	1091.8	818.9	1364.8	2456.6	1641.7	816.2	853.3

**Table 4:** Fitting parameters and $R^{2}$ for the polynomial fitting for mortar scale and $Q_{\infty }$ and $R^{2}$ for paste scale.

Composition	Mortar				Paste	
	*a* [h·J/g]	*b* [h^1/2^·J/g]	$Q_{\infty }$ [J/g]	*R^2^* [/]	$Q_{\infty }$ [J/g]	*R^2^* [/]
S05M2	1757.3	926.9	209.0	0.9991	220.2	0.9984
S05M8	3110.5	1410.4	323.0	0.9973	346.0	0.9954
S08M2	2206.6	1297.2	292.0	0.9997	278.6	0.9990
S08M8	5166.6	2184.4	418.4	0.9982	368.4	0.9958

**Table 5:** Fitting parameters and error for exponential fitting.

Composition	$Q_{1}$ [J/g]	$\tau_{1}$ [h]	$a_{1}$ [/]	$Q_{2}$ [J/g]	$\tau_{2}$ [h]	$a_{2}$ [/]	$Q_{\infty }$ [J/g]	*S* [J/g]
M-S05M2	69.37	5.91	1.08	195.64	120.67	0.34	265.01	20.81
M-S05M8	245.41	6.35	0.63	77.54	267.66	0.96	322.96	70.36
M-S08M2	35.22	5.36	1.63	268.91	33.34	0.47	304.13	14.26
M-S08M8	265.42	6.27	0.65	187.06	340.63	0.60	452.48	27.17

**Table 6:** Autogenous swelling and shrinkage of each mortar composition compared to the paste compositions.

Composition	Mortar		Paste	
	Autogenous	Autogenous	Autogenous	Autogenous
	Swelling [µm/m]	Shrinkage [µm/m]	Swelling [µm/m]	Shrinkage [µm/m]
S05M2	12.23	217.32	0	836.36
S05M8	7.64	212.73	0	1194.65
S08M2	43.51	7.58	36.55	133.61
S08M8	42.44	0	214.06	202.71

**Table 7:** Shrinkage differences between Sand 1 and Sand 2.

	Difference [%]								Standard Deviation
									[µm/m or µm/m/°C]
Equivalent Age [h]	25	50	75	100	150	200	250	300	Maximum
Autogenous strain	−36.8	−10.4	−11.3	−11.0	−5.9	−3.4	−0.3	3.1	7.5
CTE	−15.9	−6.5	−5.8	1.0	−0.2	1.8	5.2	3.5	1.8

**Table 8:** RMSE between the tensile strength and the predicted tensile strength of the different methods. mod = modified equation.

Method	RMSE
EC 2	0.51
EC 2 mod	0.15
ACI 318	0.83
ACI 318 mod	0.23

**Figure A1:**
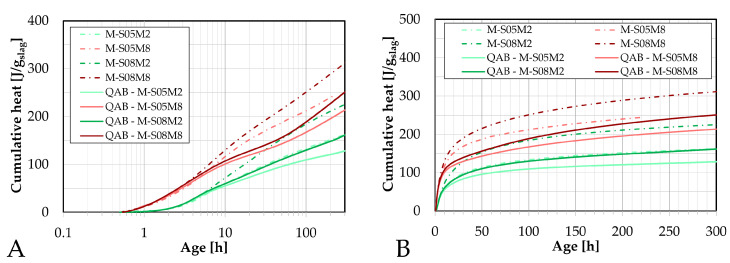
Quasi-adiabatic calorimeter results on AAS mortar compared with isothermal calorimetry results on AAS mortar, as a function of the age: (**A**). logarithmic Y-axis, (**B**). linear Y-axis.

**Figure A2:**
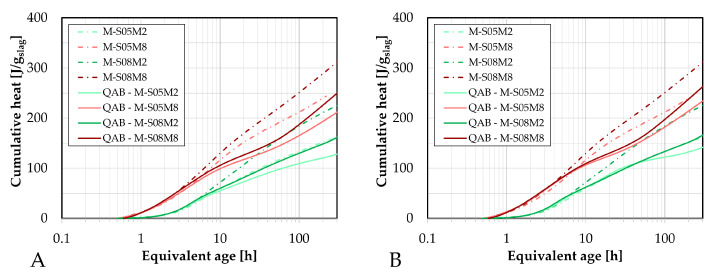
Cumulative heat of AAS mortars obtained with QAB calorimeter as function of the equivalent age computed with: (**A**). a constant activation energy over time, (**B**). a cumulative heat dependent apparent activation energy.

**Figure A3:**
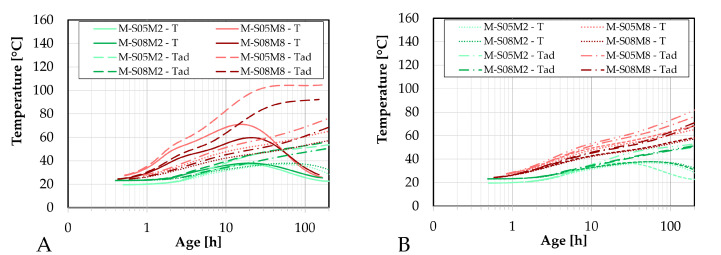
Quasi-adiabatic temperature (T) measured during the QAB test and deduced adiabatic temperature (T_ad_) as a function of: (**A**). the age of the material compared to the equivalent age computed with a constant apparent activation energy over time (eq age 1) and (**B**). the equivalent age computed with a constant apparent activation energy over time (eq age 1) compared to the equivalent age computed with a cumulative heat-dependent apparent activation energy (eq age 2). Legend: − T(age), −− T_ad_(age), ·· T(eq age 1), −·− T_ad_(eq age 1), −− T(eq age 2),−··− T_ad_(eq age 2).

**Figure A4:**
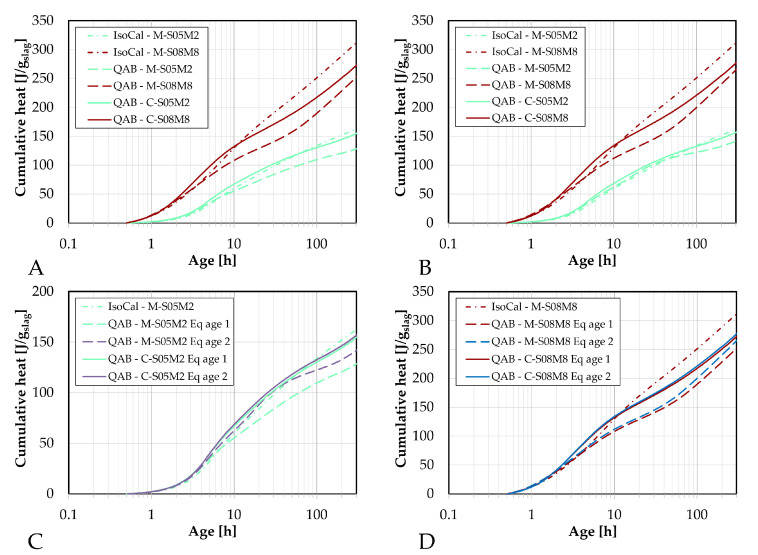
Cumulative heat of AAS concretes determined with QAB compared to mortar compositions (QAB and isothermal calorimetry) as a function of: (**A**). the eq age 1 for all compositions, (**B**). the eq age 2 for all compositions, (**C**): the eq age 1 and 2 for the S05M2 compositions, (**D**): the eq age 1 and 2 for the S08M8 compositions. Eq age 1 = equivalent age determined by the method of superposition (constant $E_{a}$ over time); Eq age 2 = equivalent age determined by the method of rates (variable $E_{a}$ over time).

## Data Availability

The original contributions presented in this study are included in the article. Further inquiries can be directed to the corresponding author.

## Abbreviations

The following abbreviations are used in this manuscript:

AAM	Alkali-activated materials
CTE	Coefficient of thermal expansion
DOR	Degree of reaction
M	Concentration in mol/L
PC	Portland cement
q	Heat flow
Q	Cumulative heat
S/B	Solution-to-binder mass ratio

## Appendix A. Quasi-Adiabatic Calorimeter

### Appendix A.1. Method

The TAM Air isothermal calorimeter serves to follow the heat release of pastes and mortars. However, it can not be used for the concrete scale as the ampoules are too small compared to the aggregates sizes. Therefore, interest has been given to the Quasi-adiabatic calorimeter, also called QAB [86].

The test consists in monitoring two 16 cm by 32 cm cylindrical samples: one compensation specimen which is considered inert and the sample to be tested. During the test, the temperature of the reacting sample, the temperature of the inert sample as well as the temperature in the lab are measured [86].

During the test, a part of the heat released by the reaction of the material increases the temperature of the specimen. A second part increases the temperature of the calorimeter and the last part is discharged to the outside. The heat released can thus be computed in the following way [86]:

(A1) $$Q\left(t\right)=C_{tot}\;\cdot \;\left(T_{sample}\left(t\right)-T_{sample}\left(0\right)\right)+\alpha_{hc}\int_{0}^{t}\theta \left(t\right)dt$$

where

(A2) $$C_{tot}=C_{cal}+C_{sample}=C_{cal}+\left(\mu_{s}\;\cdot \;\left(m_{c}+m_{s}+m_{g}\right)+\mu_{l}\;\cdot \;m_{l}\right)$$

with

- $C_{sample}$: heat capacity of the material [J/°C]. This is computed following Equation (A2).
- $\mu_{s}$: specific heat capacity for solids in the composition (cement, sand and aggregates) [J/°C/kg]. This has been taken equal 800 J/°C/kg [86].
- $\mu_{l}$: specific heat capacity for the solution [J/°C/kg]. This has been evaluated based on value in the literature and is explained later in this section.
- $m_{b}$, $m_{s}$, $m_{g}$, $m_{l}$: mass of the binder, sand, coarse aggregates and solution in the composition, respectively.
- $C_{cal}$: heat capacity of the calorimeter [J/°C]. This was obtained from the calibration step performed prior to this thesis.
- $C_{tot}$: total heat capacity which is the summation of the heat capacity of the material and that of the calorimeter.
- *t*: time elapsed since the start of the mixing [h].
- $T_{sample}$: temperature of the fresh sample [°C].
- $T_{ext}$: exterior temperature [°C];
- $T_{control}$: temperature of the inert (control) specimen [°C].
- Q(t): heat released at time t since the start of the test [J].
- θ(t): temperature deviation between the fresh sample and the control specimen at time t ($\theta \left(t\right)=T_{sample}-T_{control}$) [°C].
- $\alpha_{hc}$: heat conduction coefficient [J/h/°C]. This was obtained from the calibration step performed prior to this thesis.
- $m_{b}$: mass of the fresh material cast into the cardboard mold.

Finally, to be able to compare to the isothermal calorimetry two extra steps are added. First, the obtained cumulative heat is divided by the mass of blast-furnace slag in order to have the normalized cumulative heat [J/g_slag_]. The second step is to take into account the maturity of the material. In fact, as the temperatures have increased up to 73 °C in M-S05M8, the maturity of the material is much higher at time t than if the temperature had been kept at 20 °C during the same time t.

The specific heat capacity of the solution has been evaluated based on a literature search. Unfortunately, not a lot of data concerning the specific heat capacity is available. Values for a 2 M [87], 6 M [88] and a 9.9 M [88] NaOH solution have been found. In addition, the specific heat capacity of water, corresponding to a 0 M solution, is also known [86]. Thanks to this, the specific heat capacity of the 8 M NaOH solution could be deduced by a second degree polynomial interpolation.

Two compositions (M-S05M2 and M-S05M8) were tested twice. Consequently, the repeatability between two specimens was assessed. As a function of the equivalent age computed with the constant equivalent age, the average standard deviation is 4.35 J/g for M-S05M2 and 4.29 J/g for M-S05M8. The difference is quite similar for both compositions. The standard deviation was also assessed for the curves put as a function of the equivalent age computed with a variable equivalent age. The resulting average standard deviation is 1.78 J/g for M-S05M2 and 2.46 J/g for M-S05M8. Even though the reduction for the M-S05M8 compositions is less high, the standard deviation is still nicely reduced. This shows that in the case where higher temperatures are reached during the test, a variable apparent activation energy should be used.

### Appendix A.2. Results and Discussion on Mortar

The reaction kinetics of AAS mortars were also investigated using a quasi-adiabatic (QAB) calorimeter. The results are presented in Figure A1, plotted as a function of age. Initially, the heat release curves for all compositions are superimposed, similar to what was observed for paste compositions with the same molar concentration [20]. These curves separate at some age, although this point of separation does not appear to be strongly dependent on the testing scale or method.

Following this separation, the overall heat release measured with the QAB method is consistently lower than that obtained using isothermal calorimetry. A similar trend was noted by Carette [23] for Portland cement-based systems. However, the difference was less pronounced compared to that observed for AAS mortars.

The QAB method involves monitoring the internal temperature of a 16 cm × 32 cm cylindrical specimen. Some of the differences in results might be related to variations in the mixing procedure: a small 1 L mixer is used for isothermal calorimetry while a larger 12 L mixer is used for the QAB test. These different mixing scales could affect the dispersion of the grains and the homogeneity of the mix.

Moreover, temperature effects present in the QAB method may contribute to the discrepancies. In this analysis, the results are presented as a function of the equivalent age, which takes into account the temperature-dependent maturity using the activation energy previously determined on the paste scale. It should be noted that a constant activation energy is assumed throughout the test, independently of the actual temperature variations. In reality, the activation energy is both time and temperature-dependent.

The curing temperature influences the reaction kinetics of these materials [71]. Given that temperatures of maximum 73 °C were reached during the QAB test, it is likely that the reaction kinetics were affected.

The computation of the equivalent age has also been done differently. The apparent activation energy can be considered as being constant over the duration of the test and across the temperature range. However, this does not reflect fully the reality. In fact, the apparent activation energy is time and temperature-dependent [86]. Therefore, the apparent activation energy has also been assessed with the method of rates between 20 °C and 30 °C, depending on the cumulative heat produced. The results are shown in Figure A2B. The temperature measured during the tests used for the computation of the cumulative is shown in Figure A3 as function of the age of the material (A), the equivalent age computed with a constant apparent activation energy (A and B) and the equivalent age computed with a variable apparent activation energy (B). Based on the temperature measured in quasi-adiabatic conditions, the temperature in adiabatic conditions can be computed, see Equation (A3). These temperatures are also represented in the same figure.

(A3) $$T_{ad}\left(T\right)=T_{concrete}\left(0\right)+\frac{Q(t)}{C_{material}}$$

In general, the second method of computing the equivalent age yields slightly higher cumulative heat results. However, the results are still relatively different from the isothermal calorimetry results. The curves of the compositions activated by the same concentration stay overlapped longer. Specifically, the overlap for the 2 M curves lasts about 20 h and about 10 h for the 8 M compositions.

It is important to note that temperatures exceeding 30 °C can be reached during quasi-adiabatic testing. Therefore, evaluating the apparent activation energy at higher temperature ranges could provide more accurate equivalent age computations and potentially improve the correlation with isothermal calorimetry data.

### Appendix A.3. Results and Discussion on Concrete

The cumulative heat of the AAS concrete compositions has been evaluated with the quasi-adiabatic calorimeter. The results are shown in Figure A4.

The age of the samples tested with the QAB calorimeter is converted into the equivalent age in order to account for the non-isothermal conditions and the consequent evolution of the maturity over time. The first method involved calculating the equivalent age using the apparent activation energy, which was assumed to be constant throughout the testing period and the temperature variations. This approach is represented in Figure A4A. In Figure A4C,D, it is represented by the green and red-colored curves, respectively. These results were then compared to the isothermal calorimetry data obtained for the mortars. Overall, the cumulative heat results at the concrete scale were found to be closer to the isothermal calorimetry results than those from the mortar scale QAB measurements.

A second method was also explored, which considers an apparent activation energy that varies depending on the cumulative heat. This approach, based on the rate method explained in Lacante et al. [44], was applied. These results can be found in Figure A4B. In Figure A4C,D, it is represented by the purple and the blue-colored curves, respectively. For both M-S08M8 and C-S08M8, the difference between the two methods is minimal, with the variable activation energy method yielding slightly higher cumulative heat values. A similar trend is observed for M-S05M2. In the case of C-S05M2, the results do almost overlap with the isothermal calorimetry results. At the concrete scale, the two equivalent age computation methods yield very similar results, with only a slight increase in cumulative heat (3.34 ± 1.58 J/g_slag_) compared to the mortar scale (13.54 ± 0.18 J/g_slag_). In conclusion, even though the variation of the apparent activation energy is taken into account, the isothermal calorimetry method and the quasi-adiabatic calorimeter method do not yield the same results, except for the C-S05M2 composition. Interestingly, an initial overlap between the mortar isothermal calorimetry, mortar QAB, and concrete QAB curves can be observed for each composition. This overlap lasts longer for the S05M2 compositions (around four hours) compared to the S08M8 compositions (about two hours).
